# Dynamic Interactions and Deformation Mechanisms in Nanocutting of Carbon Nanotube Reinforced Ni-Based Composites

**DOI:** 10.3390/nano16191226

**Published:** 2026-09-28

**Authors:** Ping Zhang, Zhimin Zhao, Hui Yang, Junhong Guo, Youqiang Wang

**Affiliations:** 1 College of Intelligent Manufacturing, Qingdao Huanghai University, Qingdao 266520, China; 2 College of Mechanical Engineering, Guangdong Ocean University, Zhanjiang 524088, China; 3 School of Materials Science and Engineering, Jiamusi University, Jiamusi 154007, China; 4 School of Materials Science and Engineering, Inner Mongolia University of Technology, Hohhot 010080, China; 5 College of Intelligent Manufacturing, Qingdao University of Technology, Qingdao 266520, China

**Keywords:** carbon nanotubes, nanocutting, subsurface defects, dislocation evolution, molecular dynamics simulations

## Abstract

Molecular dynamics simulations were employed to investigate the effects of cutting speed and cutting depth on the nanocutting behavior of carbon nanotube (CNT)-reinforced Ni composites. The results show that increasing cutting speed from 50 to 200 m/s reduces the average tangential cutting force by approximately 16.5%, while the cutting temperature increases by 26.7% at a cutting distance of 200 Å. Cutting depth produces a stronger influence on subsurface deformation, with deeper cutting accompanied by increased stress localization, structural disorder, and dislocation activity. In contrast, dislocation evolution exhibits a non-monotonic dependence on cutting speed, indicating that thermal and mechanical effects act concurrently under different cutting conditions. Pronounced changes in local deformation and dislocation behavior are also observed near the CNT–matrix region. These results characterize the coupled thermomechanical and defect responses of the selected CNT–Ni system under different nanocutting conditions.

## 1. Introduction

Carbon nanotubes (CNTs) have garnered extensive attention in the field of composite materials owing to their extraordinary mechanical strength, high aspect ratio, and superior thermal and electrical conductivity. When integrated as a reinforcing phase within metallic matrices, CNTs can markedly enhance the mechanical integrity of the composites [1,2,3], leading to improvements in hardness, yield strength, fatigue resistance, and fracture toughness. This makes CNT-based metal matrix composites (MMCs) highly promising candidates for use in advanced engineering applications, particularly in aerospace, automotive [4,5], and microelectromechanical systems (MEMS). The synergistic strengthening effect arises primarily from the load transfer capability of CNTs, their ability to impede dislocation motion, and their influence on crack propagation resistance [6].

In recent years, substantial progress has been made in understanding the structural and mechanical behavior of CNT-reinforced MMCs based on various matrix systems, such as Fe, Ti, Cu, Mg, and Ni. For example, Faria et al. [7] employed molecular dynamics (MD) simulations to evaluate the mechanical responses of CNT-reinforced Fe and Ti nanocomposites under different loading conditions. Their results emphasized the critical roles of CNT alignment and buckling behavior in modulating the plastic deformation mechanisms of the metallic matrix. Similarly, Wu et al. [8] investigated interfacial characteristics between CNTs and Ti, demonstrating that the shear strength and peak load-carrying capacity at the interface reached up to 37.8 MPa and 245 nN, respectively—highlighting the significant reinforcement potential at the nanoscale. Despite their favorable mechanical properties, CNT/MMCs still face challenges associated with CNT dispersion and interfacial bonding [9,10,11]. Ni is a promising matrix for CNT reinforcement because of its favorable compatibility with carbon-based nanomaterials and its ability to form relatively stable CNT–Ni interfaces [12,13,14,15]. Recognizing this, researchers such as Yan et al. [16] have focused on the mechanical performance of CNT/Ni composites. Their MD simulations revealed that the mechanical properties of the composite material were highly sensitive to the orientation and volume fraction of CNTs. Interestingly, as the CNT rotation angle varied from 0° to 90°, the tensile properties initially deteriorated and then improved, indicating a non-linear dependence of mechanical behavior on CNT alignment. The incorporation of CNTs also increases the complexity of precision machining because material removal involves localized CNT–matrix interactions and heterogeneous stress fields [17,18,19]. At the nanoscale, dislocation activity, lattice distortion, interfacial sliding, and thermomechanical coupling become important features of the cutting response. Molecular dynamics (MD) simulations provide an effective approach for resolving these atomistic mechanisms [20]. Recent studies have demonstrated the utility of MD simulations in nanomachining research. For instance, Fang et al. [21,22] examined high-temperature nanocutting behavior in NiTi shape memory alloys, revealing that elevated temperatures facilitated dislocation movement and reduced residual stress accumulation. In another study, Zhang et al. [23] investigated the influence of tool radius and cutting parameters on the nanomachining of FeCoNiCrAl_0_._6_ high-entropy alloys, and found that dislocation density increased sharply when the tool radius dropped below 7.5 Å and cutting velocity reached 150 m/s—highlighting the sensitivity of cutting response to nanoscale geometric factors.

Despite extensive research into the bulk mechanical behavior of CNT-reinforced metal composites, investigations into their machinability, particularly under nanocutting conditions, remain relatively limited. The highly complex interfacial interactions between CNTs and metallic matrices during cutting introduce additional challenges that conventional theories or empirical models struggle to address. The lack of systematic understanding of the coupled effects of cutting parameters and CNT–matrix interactions during nanocutting has hindered a comprehensive understanding of the deformation and material removal mechanisms in CNT/Ni systems. To bridge this gap, the present study employs advanced molecular dynamics simulations to explore the deformation behavior and interfacial interactions of CNT-reinforced Ni composites during nanocutting [24,25]. By systematically varying the cutting speed and cutting depth, this study investigates their effects on cutting forces, temperature evolution, stress–strain responses, chip morphology, subsurface defects, atomic structure, and dislocation evolution in CNT-reinforced Ni composites. The CNT configuration is kept fixed in the present study so that the influence of the two principal cutting parameters can be examined under consistent reinforcement conditions. The findings of this study are expected to provide atomic-scale insights into the nanocutting behavior of CNT/Ni composites and serve as a reference for further studies on cutting-parameter effects and nanocomposite design.

## 2. Model and Methods

### 2.1. Model and Parameters

In this study, we used the open-source software Atomsk (v 0.13) to generate a single-crystal Ni matrix material and inserted a 60 Å-long armchair (4,4) CNT into the Ni matrix to construct a CNT-Ni composite material. To ensure a smooth simulation process, we used molecular dynamics (MD) software LAMMPS (22 July 2025—Update 1) to perform atomic-level assembly of the CNT and Ni matrix, removing overlapping atoms within a 3 Å radius in the assembly region, resulting in the final CNT-Ni composite material model. The CNT-Ni composite nanocutting MD simulation model is shown in Figure 1.Throughout the simulation series, the geometry, orientation, and spatial configuration of the CNT were kept unchanged, while cutting speed and cutting depth were varied as the principal process parameters. During the cutting process, the deformation behavior, subsurface defects, dislocation evolution, and other microscopic phenomena of the CNT-Ni composite material were analyzed using the visualization software OVITO (v 3.14), enabling a comprehensive understanding of the microscopic structural changes during the cutting process [26]. The thickness of the Newton and thermostat layers was set to 10 Å, with the temperature of the thermostat layer controlled at 293 K. The workpiece was divided into fixed, thermostat, and Newtonian regions. The fixed atoms were used to maintain the structural stability of the workpiece, the thermostat atoms served as a heat-dissipation layer, and the Newtonian atoms represented the dynamically evolving cutting region. The Berendsen thermostat was applied to the thermostat layer to maintain its temperature at 293 K and to dissipate the heat generated during the cutting process. Atoms in the Newton layer followed Newton’s second law, and the velocity-Verlet algorithm was employed for numerical integration to ensure the stability and reliability of the simulation results. Periodic boundary conditions were applied along the Y direction, while non-periodic boundary conditions were imposed along the X and Z directions to accommodate the free surface and material deformation during the nanocutting process. Before starting the cutting simulation, energy minimization was applied to the workpiece using the conjugate gradient method to eliminate stress concentrations from the initial atomic configuration and ensure the system reached a stable ground state [27]. Prior to cutting, the movable atoms of the workpiece were relaxed under the NVT ensemble at 293 K for 50 ps to establish the initial equilibrium state. During the subsequent cutting process, the atoms in the Newtonian region evolved under the NVE ensemble, while the thermostat layer was maintained at 293 K using the Berendsen thermostat to dissipate the heat generated during cutting. Table 1 lists the specific parameters for the CNT-Ni composite nanocutting simulation [28,29].

### 2.2. Interaction Potential

In molecular dynamics (MD) simulations, the accuracy and reliability of the predicted physical behavior are highly dependent on the selection of an appropriate interatomic potential, which governs the nature of atomic interactions within the system. In the present investigation, the embedded atom method (EAM) potential parameterized by Mishin et al. [30] is adopted to model the atomic interactions within the single-crystal nickel (Ni) matrix. This potential is particularly suitable for metallic systems as it effectively captures the many-body interactions and electron density effects characteristic of transition metals. The mathematical form of the EAM potential employed is expressed as follows:

(1) $$E_{i}=F_{\alpha }\left(\sum_{j\neq i}\rho_{\beta }(r_{ij})\right)+\frac{1}{2}\sum_{j\neq i}\phi_{\alpha \beta }\left(r_{ij}\right)$$

where E_i_ represents the total system energy, Fα is the embedding energy related to the electron density ρ_β_, ϕ is the interatomic potential, and r_ij_ is the distance between atoms i and j.

To accurately model the covalent bonding characteristics between carbon atoms within the diamond cutting tool, the Tersoff potential is utilized in this study to define the C–C interatomic interactions. As a well-established potential function for covalently bonded systems, the Tersoff potential [31] effectively captures the directionality and bond-order dependence inherent to carbon-based materials. The mathematical formulation of the Tersoff potential employed in this simulation is given as follows:

(2) $$U=\frac{1}{2}\sum_{j\neq i}f_{c}\left(r_{ij}\right)\left[f_{R}\left(r_{ij}\right)+b_{ij}f_{A}\left(r_{ij}\right)\right]$$

(3) $$f_{R}(r_{ij})=Aexp\left(-\lambda r_{ij}\right),f_{A}\left(r_{ij}\right)=-B\exp\left(-\mu r_{ij}\right)$$

(4) $$\left.f_{c}\left(r_{ij}\right)=\left\{\begin{array}{c} 1r_{ij}<R-D\\ \frac{1}{2}-\frac{1}{2}sin\left[\frac{\pi }{2}\frac{r_{ij}-R}{D}\right]\\ 0r_{ij}>R+D \end{array}\right.\right.\left|R-r_{ij}\right|\leq D$$

where *fc*(*r_ij_*) is the cutoff function for atomic potential, *f_R_*(*r_ij_*) and *f_A_*(*r_ij_*) are the repulsive and attractive components of the potential, *b_ij_* is a modulation factor, and *r_ij_* is the interatomic distance. *R* and *D* represent the cutoff region’s position and width.

The adaptive intermolecular reactive empirical bond order (AIREBO) potential [32] is widely recognized for its versatility in modeling both bonded and non-bonded interactions in carbon-based nanostructures, including carbon nanotubes (CNTs) and graphene. In this study, the AIREBO potential is employed to characterize the C–C atomic interactions within CNTs, effectively capturing the bond-breaking and bond-forming processes essential for simulating their mechanical response under deformation.

(5) $$E=\frac{1}{2}\sum_{i}\sum_{j\neq i}\left[E_{ij}^{REBO}+E_{ij}^{LJ}+\sum_{k\neq i,j}\sum_{l\neq i,j,k}E_{kijl}^{tors}\right]$$

where V_bond_ represents the covalent bonding interactions, V_nonbond_ represents non-bonded interactions, and V_torsion_ represents torsional interactions.

To accurately describe the weak van der Waals interactions between carbon atoms in the carbon nanotubes (CNTs) and nickel atoms in the metallic matrix, the Lennard-Jones (L-J) potential [16] is employed in this study. The parameters required for the L-J potential were adopted from previous atomistic simulations of CNT/Ni systems [33]. This potential function is particularly suitable for modeling non-bonded interfacial interactions. The mathematical form of the Lennard-Jones potential utilized in this work is expressed as follows:

(6) $$E=4\varepsilon \left[{\left(\frac{\sigma }{r}\right)}^{12}-{\left(\frac{\sigma }{r}\right)}^{6}\right]\left(r<r_{c}\right)$$

(7) $$\varepsilon =\sqrt{\varepsilon_{1}\varepsilon_{2}}$$

(8) $$\sigma =\frac{\left(\sigma_{1}+\sigma_{2}\right)}{2}$$

where *ϵ* is the depth of the potential well, and *σ* is the finite distance at which the interatomic potential is zero.

The adopted Ni–C Lennard–Jones parameterization has been previously applied to CNT/Ni systems [34], for which an equilibrium CNT–Ni separation of approximately 2.45 Å was reported. This value is within the characteristic range reported for Ni–C interfaces, supporting the use of the selected parameterization for describing the non-bonded interaction between the CNT and Ni matrix. It should be noted that the Lennard–Jones potential employed here provides an effective description of the physical CNT–Ni interaction rather than a reactive representation of interfacial chemical bonding. Therefore, the present interface model is primarily used to capture the local mechanical interaction between the CNT and Ni matrix during nanocutting. Accordingly, quantitative interpretations of interface-sensitive responses, particularly local force transfer and defect evolution near the CNT–Ni interface, should be considered within the approximation of the present non-reactive interface model.

The Morse potential is employed to characterize the interaction forces between carbon atoms in the diamond cutting tool and nickel atoms in the workpiece. The corresponding Ni–C interaction parameters were adopted from the nanoscale cutting study of Hao et al. [35]. This potential is suitable for describing the attractive and repulsive interactions at the tool–workpiece interface during nanocutting. The functional form of the Morse potential applied in this simulation is given by

(9) $$E=D_{0}\left[e^{-2\alpha (r-r_{0})}-2e^{-\alpha (r-r_{0})}\right]\;\;\;\;\;\;\;\;\;\;\;\;\;\;\;\;\;\;\;r<r_{c}$$

where *D*_0_ is the binding energy, *α* is the elasticity coefficient, and *r*_0_ is the equilibrium bond distance, with *r_ij_* representing the atomic separation.

Since the diamond cutting tool was treated as a rigid body, the non-bonded interaction between the carbon atoms of the tool and those of the CNT was described using the Lennard–Jones potential. The corresponding interaction parameters were adopted from Ref. [34].

The interatomic potentials and corresponding parameters used for the different atomic interactions are summarized in Table 2.

### 2.3. Energy Dissipation Mechanisms During Nanocutting

During the nanocutting process, the mechanical work introduced by the cutting tool can be redistributed through several concurrent mechanisms, including elastic deformation, irreversible atomic rearrangement, defect formation, interfacial interaction, and heat generation. For CNT-reinforced metallic composites, these processes are also affected by the mechanical response of CNTs and their interaction with the surrounding Ni matrix. Previous studies have shown that CNTs subjected to complex loading can exhibit elastic deformation, local structural rearrangement, and interfacial interaction, which may influence the transfer and redistribution of mechanical energy within the composite system [37,38,39].

At relatively small deformation levels, CNTs can store part of the mechanical energy through elastic deformation owing to their high stiffness and nonlinear elastic response [31]. The constitutive relationship describing this elastic response can be expressed as

(10) $$\sigma =\sum_{i=0}^{n}E_{i}\varepsilon^{i}$$

where *E* is the elastic constant of CNTs, and *ε* is the strain. The relationship between the stress and strain tensors can be expressed as

(11) $$\sigma :\varepsilon =\sum_{i=0}^{n}E_{i}\varepsilon^{i}:\varepsilon$$

Accordingly, the energy associated with elastic deformation can be written as

(12) $$E_{e}=\int_{\Omega }\sum_{i=0}^{n}E_{i}\varepsilon^{i}:\varepsilon dV$$

These expressions describe the fundamental relationship between CNT deformation and elastic energy storage and release.

As the cutting load increases, irreversible deformation becomes increasingly important and is accompanied by atomic rearrangement, local structural disorder, defect formation, and dislocation evolution [40]. These processes contribute to the conversion and redistribution of mechanical energy within the deformation zone. In CNT–Ni composites, the presence of the CNT and its interaction with the surrounding matrix can further influence local deformation pathways, stress localization, and defect evolution [41].

Interfacial interaction and relative motion among the cutting tool, CNT, and Ni matrix constitute another important pathway of energy redistribution during nanocutting. Local contact, sliding, and frictional interaction can alter the mechanical response of the cutting region and contribute to energy transfer across the interface [42]. In general, the work associated with frictional interaction can be expressed as

(13) $$E_{f}=\int_{\Omega }\mu \left(\varepsilon_{c}\right)F_{N}v_{cut}dA$$

This formulation captures the energy redistribution associated with frictional interaction and relative motion at the interface.

During the cutting process, part of the mechanical work is converted into thermal energy through plastic deformation, atomic vibration, and tool–workpiece interaction. The generated heat is subsequently transferred from the highly deformed cutting region toward the surrounding material. From a continuum perspective, this heat-transfer process can be described by the general heat-conduction relation:

(14) $$Q=-k_{C}\nabla T+\rho C_{p}\varepsilon_{r}\Delta T$$

where *k* is the thermal conductivity, *ρ* is the material density, *C_p_* is the specific heat capacity, *ε_r_* is the strain rate, and ∇*T* is the temperature gradient. This relation characterizes the heat-transfer process associated with thermal energy redistribution during nanocutting.

Based on these considerations, energy dissipation during nanocutting can be regarded as a coupled process involving elastic deformation, irreversible atomic rearrangement, defect evolution, interfacial interaction, and heat generation. Collectively, the above formulations establish a theoretical framework for understanding the principal pathways of energy storage, transfer, conversion, and redistribution in the CNT–Ni nanocutting system.

### 2.4. Post-Processing and Calculation of Output Quantities

The atomic trajectories generated by the MD simulations were post-processed using OVITO [43]. The cutting force was obtained from the total force acting on the cutting tool, with $F_{x}$ and $F_{z}$ representing the tangential and normal components, respectively. The average cutting forces were calculated over the steady-state cutting stage indicated in the corresponding force–distance curves. The steady-state cutting stage was defined as the period after the initial tool-entry transient, during which the cutting force fluctuated around a relatively stable mean value. The cutting temperature was evaluated for atoms within the Newtonian region of the workpiece.

The per-atom stress tensor was calculated in LAMMPS using the stress/atom compute [44]. Since the output of stress/atom represents a stress–volume quantity, the six tensor components were normalized by the corresponding atomic Voronoi volume obtained using the voronoi/atom compute [37]. The resulting stress components, $\sigma_{xx}$, $\sigma_{yy}$, $\sigma_{zz}$, $\sigma_{xy}$, $\sigma_{xz}$ and $\sigma_{yz}$ were converted from bar to GPa. The local von Mises equivalent stress was then calculated as

(15) $$\sigma_{VM}=\sqrt{\frac{1}{2}\left[{\left(\sigma_{xx}-\sigma_{yy}\right)}^{2}+{\left(\sigma_{yy}-\sigma_{zz}\right)}^{2}+{\left(\sigma_{xx}-\sigma_{zz}\right)}^{2}+6\left(\sigma_{xy}^{2}+\sigma_{yz}^{2}+\sigma_{xz}^{2}\right)\right]}$$

Atomic shear strain was calculated using the Atomic Strain modifier implemented in OVITO [43], with the relaxed atomic configuration prior to cutting taken as the reference configuration. The resulting atomic shear-strain field was used to characterize strain localization and shear-band evolution. The shear-band angle was defined as the angle between the principal direction of the strain-localized region and the cutting direction, while the shear-band width was measured normal to the principal shear-band direction. The subsurface defect-layer thickness was determined from the depthwise extent of the continuous defect-rich region beneath the machined surface.

Local atomic environments were identified using the Common Neighbor Analysis (CNA) method implemented in OVITO [43,45]. According to their local coordination environments, Ni atoms were classified as FCC, HCP, BCC, or other local atomic environments. The same structural-identification criterion was consistently applied to all simulated cutting conditions to enable comparison of the evolution of local atomic structures.

Dislocation structures were identified using the Dislocation Extraction Algorithm (DXA) implemented in OVITO [33,43]. The extracted dislocations were classified according to their Burgers vectors as 1/2<110> (Perfect), 1/3<100> (Hirth), 1/3<111> (Frank), 1/6<110> (Stair-rod), and 1/6<112> (Shockley) dislocations. The dislocation density was calculated from the total length of the extracted dislocation lines divided by the corresponding analysis volume:

(16) $$\rho_{d}=\frac{L_{d}}{V}$$

where $L_{d}$ is the total dislocation-line length and V is the analysis volume. Accordingly, the dislocation density has dimensions of inverse area and is expressed in Å^−2^ or the present atomic-scale model.

The comparisons presented in this study are based on individual MD trajectories obtained under identical simulation protocols for each cutting condition. Therefore, the reported variations are interpreted as comparative atomistic trends rather than statistically significant differences. Ensemble-based uncertainty analysis using independent initial velocity seeds was not performed in the present study.

## 3. Results and Analysis

### 3.1. Cutting Mechanics and Temperature Evolution

During the nanocutting process, the evolution of cutting force and cutting temperature serves as a critical indicator of the material’s plastic flow behavior, defect nucleation and propagation, and structural transformation at the atomic scale. These parameters reflect the complex interplay between mechanical load, thermal effects, and interfacial phenomena. Figure 2 presents the fluctuation in cutting force as well as the corresponding defect distributions observed during the steady-state cutting phase under various cutting speeds. As the cutting velocity increases, there is a noticeable amplification in the fluctuation amplitude of the cutting force, accompanied by a significant transformation in defect evolution patterns. At a cutting speed of 50 m/s, the cutting force remains relatively stable, indicating a quasi-steady plastic deformation process. The defects generated are predominantly associated with grain refinement mechanisms in the chip region, where dislocation interactions and lattice rearrangements dominate. As the speed increases to 150 m/s, the shear zone experiences intensified atomic disorder, particularly in high-stress regions, where localized shear stress initiates partial amorphization. This is attributed to the elevated strain rate and enhanced friction-induced heating, which collectively disrupt the long-range crystalline order. When the cutting speed reaches 200 m/s, the proportion of atoms exhibiting disordered local environments increases markedly, particularly in the chip region. This behavior indicates severe local lattice distortion and loss of FCC ordering under the combined action of high strain rate and thermomechanical loading. The accelerated thermal softening at such high speeds facilitates plastic flow while simultaneously reducing resistance to cutting. Moreover, a distinct increase of 41% in the length of the steady-state cutting zone is observed as the cutting speed increases from 50 m/s to 200 m/s. This extension signifies a more stable and continuous material removal process at higher velocities. The combined effects of elevated temperature and concentrated shear stress enhance the material’s ductility, reduce its resistance to deformation, and lower the average cutting force. These findings indicate that higher cutting speeds modify the mechanical response of the material and the local thermomechanical conditions during nanocutting.

Figure 3 illustrates the impact of varying cutting depths on cutting force fluctuations and defect formation. As the cutting depth increases, the cutting force initially shows intensified fluctuations, followed by a gradual decrease. The most pronounced instability is observed at a depth of 15 Å, which corresponds to a critical point where the contact area between the diamond tool and the carbon nanotube (CNT) reinforcement becomes substantial. Due to the inherent stiffness and strength of CNTs, their engagement introduces high resistance and causes localized stress concentrations, thereby amplifying mechanical instability and force fluctuations. Interestingly, when the cutting depth is increased further to 20 Å, the fluctuation amplitude of the cutting force diminishes. This counterintuitive result is associated with the stabilization of the stress distribution in the shear zone. At this depth, although the tool–CNT interaction is maximized, the system enters a regime of more uniform plastic deformation due to the wider shear band and energy dissipation path. Furthermore, as cutting depth increases, defect density increases markedly, particularly in the subsurface region. At shallow depths (e.g., 5 Å), the limited tool–CNT contact results in predominantly elastic deformation, with minimal defect formation. However, at 20 Å, a substantial increase in contact length leads to extensive defect nucleation—including dislocations, stacking faults, and atomic vacancies—within both the matrix and reinforced phases [34]. These defects are highly localized near the tool–CNT interface, where the interaction induces severe lattice disruption. The presence of CNTs complicates the deformation process by resisting dislocation motion and introducing load transfer heterogeneities, thus contributing to a complex stress state and intensified defect evolution under deep cutting conditions.

Figure 4 presents the average cutting force trends under different cutting parameters, further clarifying the influence of speed and depth on the mechanical response. As illustrated in Figure 4a, increasing cutting speed results in a noticeable decrease in the average cutting forces in both the tangential (Fx) and normal (Fz) directions. Specifically, Fx decreases by approximately 16.5% as the speed increases from 50 m/s to 200 m/s. This reduction is associated with changes in the local thermomechanical response of the matrix at higher cutting speeds. The increase in temperature, together with the higher strain rate, facilitates localized plastic flow in the cutting zone. In addition, rapid cutting promotes lattice distortion and structural disorder near the tool–workpiece interface, which can further influence the cutting resistance. Figure 4b reveals that increasing cutting depth leads to a monotonic increase in cutting forces. When the cutting depth reaches 20 Å, the tangential force Fx increases to 1.84 times that observed at 5 Å. This nonlinear rise results from the expanded tool–workpiece contact area and the corresponding engagement with reinforcing CNTs. As more CNTs are intersected at greater depths, the effective cutting resistance rises due to their high modulus and fracture toughness. Moreover, increased depth also augments the energy required for crack initiation and propagation within the composite matrix, contributing to the elevated force requirement.

Figure 5 focuses on the evolution of cutting temperature within the Newtonian layer of the workpiece under various cutting parameters. A consistent trend is observed across all conditions: cutting temperature increases with cutting distance. Among all parameters, cutting speed exhibits a pronounced influence on the thermal response. At a cutting distance of 200 Å, the cutting temperature shows a 26.7% increase as the speed rises from 50 m/s to 200 m/s. This is a direct consequence of intensified tool–material interaction, which amplifies frictional heating and strain-induced thermal generation. High-speed cutting introduces severe plastic deformation in the shear zone, increasing atomic vibration and heat accumulation, thereby enhancing the local temperature field. The observed temperature increase reflects enhanced atomic vibration and heat accumulation in the cutting zone. Together with severe plastic deformation, the thermomechanical loading contributes to lattice distortion, defect evolution, and increased local structural disorder near the tool path. These changes affect the local mechanical response and deformation behavior of the material. As a result, high-speed cutting under thermal-mechanical coupling not only improves material removal efficiency but also alters the underlying deformation and failure mechanisms, offering new insights into the machinability of CNT-reinforced metal composites at the nanoscale.

### 3.2. Stress and Strain Responses

The spatial distribution and temporal evolution of stress and strain play a pivotal role in determining the mechanisms of plastic deformation, crack initiation and propagation, and eventual material failure during the nanocutting process. A detailed analysis of the von Mises stress distribution enables a deeper understanding of how different cutting parameters influence the internal response of the workpiece material, especially in carbon nanotube (CNT)-reinforced systems. Figure 6 presents the evolution of von Mises stress within the workpiece at various cutting speeds as the cutting distance increases. A clear trend is observed: as cutting speed rises, the stress distribution in the cutting zone undergoes significant transformation. At relatively low cutting speeds, such as 50 m/s, the von Mises stress remains uniformly distributed, with major concentrations localized within the shear band and chip surface region. This uniformity suggests a more stable and gradual material flow, where the energy input is insufficient to cause pronounced microstructural disruptions or localized overheating. However, as the cutting speed increases to 150 m/s and further to 200 m/s, the internal stress within the chip intensifies considerably, and the stress distribution becomes increasingly non-uniform and localized in the shear deformation zones. This behavior is attributed to enhanced strain rates and frictional heating, which result in elevated thermal and mechanical energy accumulation at the tool–workpiece interface. At these high speeds, rapid plastic deformation and thermal softening lead to transient material instability, causing stress to concentrate in narrow zones rather than diffusing uniformly. A notable observation at the highest speed (200 m/s) is the reduced bending deformation of CNTs embedded in the matrix. This phenomenon is attributed to two concurrent factors. First, the transient thermal response at high cutting speeds increases the internal energy of the material, which softens the matrix and reduces the transmission of shear stresses necessary to bend the rigid CNTs. Second, the inherent high stiffness and modulus of CNTs resist excessive deformation, especially when subjected to localized compressive and tensile forces during rapid shearing. As a result, the CNTs retain their structural integrity to a greater extent, even under high cutting stress, while still enhancing interfacial strength and stress transfer within the composite. Additionally, as the cutting distance reaches 200 Å, the chip height exhibits a noticeable increase with rising cutting speed. This variation reflects changes in material flow and chip morphology under different cutting-speed conditions. Combined with the observed stress distribution, the results indicate that cutting speed influences the plastic-flow behavior and deformation pathways of the material. Figure 7 further explores the distribution and intensity of von Mises stress at different cutting depths. The figure reveals that stress levels within the chip and shear zone rise with increasing cutting depth, while the spatial extent of the stress field becomes more diffused. At a cutting depth of 10 Å, the onset of substantial contact between the tool and the CNT-reinforced matrix results in an abrupt spike in localized stress. This is due to the high mechanical resistance posed by the CNTs, which induces significant stress concentrations around the contact area. When the cutting depth reaches 20 Å, the stress levels in the chip and shear zone reach their maximum. The deeper tool penetration increases the contact interface area, resulting in a greater mechanical interaction with both the metallic matrix and the CNTs. The CNTs, subjected to intense inward compressive stress, begin to deform plastically. Although CNTs possess remarkable tensile strength, their resistance to compressive loads and shear stress at the atomic scale becomes overwhelmed under extreme cutting conditions, leading to localized buckling and interfacial slippage. From a microscale mechanics perspective, the progressive increase in cutting depth leads to a proportional rise in the contact area and interaction time between the tool and the workpiece. This causes higher stress accumulation and increased non-uniformity in stress distribution across the shear zone. Furthermore, as the tool engages more CNTs at greater depths, the energy required to deform or fracture these high-strength reinforcements also increases, contributing to elevated von Mises stress levels. The rigidity of CNTs restricts uniform stress transfer, forcing stress to accumulate near the interfaces, thereby amplifying local stress gradients and mechanical instability. In terms of material response, the combined effect of high cutting speed and increased depth introduces a complex thermomechanical environment that strongly influences the deformation modes and failure mechanisms. High-speed cutting promotes strain localization and accelerates plastic flow, accompanied by pronounced lattice distortion and local structural disorder in highly deformed regions. These changes modify the local deformation response of the material, and the resulting stress–strain behavior reflects the coupled influence of mechanical loading and temperature evolution. The influence of cutting depth intensifies this effect by increasing the interaction between the tool and the embedded CNTs, leading to stronger interfacial shear stresses and higher strain energy accumulation within the matrix. At greater depths, the expanding contact zone facilitates more pronounced stress transmission across both the metallic phase and the CNT reinforcement. This introduces complex load transfer mechanisms, wherein the tool’s mechanical load is distributed among the matrix and the CNTs depending on their orientation, aspect ratio, and interfacial bonding strength. These interactions not only contribute to the overall increase in stress magnitude but also promote more homogeneous stress distribution at the macro level, while retaining local stress peaks near the interfaces. Overall, the evolution of von Mises stress during nanocutting reflects the intricate interaction between the cutting tool, matrix, and reinforcement phase. The stress field is not only indicative of the immediate mechanical response but is also closely linked to material microstructure, deformation localization, and thermal activation effects. At the nanoscale, the high rigidity and load-bearing capacity of CNTs serve to regulate the propagation of plastic zones and suppress certain modes of deformation. However, their presence also introduces stress concentration points that exacerbate instability under severe cutting conditions. These findings underscore the dual role of CNTs as both reinforcing agents and stress modulators in nanomachining processes. The insights gained from analyzing stress and strain distributions under various cutting parameters provide a fundamental understanding of the deformation mechanisms in CNT-reinforced metal composites. Such understanding is essential for optimizing cutting strategies, tool design, and process parameters to improve machining efficiency, reduce tool wear, and control defect generation in high-performance composite materials.

### 3.3. Shear Strain and Shear Band Evolution

Figure 8 illustrates the shear strain distribution within the workpiece as the cutting distance increases under varying cutting speeds. The results demonstrate that cutting speed exerts a significant influence on the morphology and evolution of the shear band. Specifically, as the cutting speed increases from 50 m/s to 200 m/s, a noticeable narrowing of the shear band is observed, along with a reduction in the shear line angle from 38.2° to 35.2°. This phenomenon reflects the fundamental changes in deformation behavior associated with high-speed machining, where material flow becomes increasingly localized, and strain is concentrated within narrower regions of the shear zone. At lower cutting speeds (e.g., 50 m/s), the deformation of the material is relatively uniform across a broader shear zone. This is attributed to moderate strain rates and limited heat generation, which allow for a more distributed plastic flow. Consequently, the shear band appears wider, and the shear line angle is higher, indicating a smoother transition between undeformed and deformed zones. However, as the cutting speed increases to 150 m/s and eventually to 200 m/s, the deformation rate of the material rises sharply. This increase in strain rate intensifies the heat generated at the tool–workpiece interface, resulting in a substantial thermal softening effect. Thermal softening lowers the yield strength of the material, facilitating more intense plastic flow, but also localizing it within narrower regions, hence reducing the shear band width and shear line angle. The observed strain localization at higher speeds suggests that plastic deformation becomes more concentrated and abrupt, forming a distinct shear band that isolates the deformation zone from the bulk material. The narrowing of the shear band at high speeds is further enhanced by the presence of carbon nanotubes (CNTs) within the matrix. While the degree of CNT bending is somewhat reduced under high-speed conditions due to their rigidity and reduced contact time, their mechanical constraint on the surrounding matrix remains pronounced. Owing to their high axial stiffness and limited deformability, CNTs resist shear-induced distortion, which in turn leads to localized shear strain gradients in the surrounding material. Moreover, CNTs restrict the lateral expansion of the shear band by acting as nanoscale reinforcement elements. Their presence prevents the shear strain from diffusing into adjacent regions, thereby enhancing strain concentration within the narrow shear zone. This mechanism underscores the dual role of CNTs in high-speed cutting: while they serve as reinforcements that improve structural integrity, they also contribute to mechanical instability by localizing deformation. These results highlight the critical role of strain localization in controlling the efficiency and quality of the cutting process and emphasize the importance of understanding how reinforcement phases influence nanoscale deformation behavior.

Figure 9 presents the shear strain distribution in the workpiece under different cutting depths, offering further insight into the evolution of strain localization and shear band structure. As the cutting depth increases from 5 Å to 20 Å, both the width of the shear band and the thickness of the plastic deformation layer increase markedly. For instance, at a cutting depth of 5 Å, the shear deformation layer near the processed surface measures approximately 7.1 Å. This value increases to 13.3 Å when the cutting depth reaches 20 Å, indicating a substantial enhancement in plastic deformation volume and strain diffusion. At shallow depths, such as 5 Å, the contact between the tool and workpiece is minimal, resulting in limited material flow and relatively minor shear strain. In this regime, shear lines are barely distinguishable, and deformation is concentrated near the surface. However, as the cutting depth increases to 15 Å, the tool engages more CNTs, and the mechanical interaction becomes more pronounced. The shear line angle increases to 34.6°, and the width of the shear band expands, signaling a more extensive material response to cutting. At 20 Å, these trends become more evident, with the shear line angle rising to 36.1°, and the plastic deformation zone extending deeper into the material. This increase in shear strain distribution with cutting depth is primarily attributed to two factors. First, greater cutting depths increase the contact area and contact duration between the tool and both the matrix and CNTs. This amplifies the mechanical load transferred into the material, leading to a more intense and widespread plastic response. Second, as more CNTs are intersected by the cutting tool, the reinforcing phase imposes greater constraint on deformation, altering the stress distribution and guiding the propagation of the shear band. At deeper cutting levels, CNTs are subjected to stronger inward compressive forces, causing them to act as rigid inclusions that oppose deformation. This restricts the lateral spread of shear strain and directs plastic flow into more confined regions. The localized interaction between the tool and the CNT-reinforced matrix introduces complex load redistribution and stress concentration phenomena. As a result, although the overall shear band becomes wider, the strain remains concentrated around the tool path and the immediate vicinity of the reinforcement–matrix interface. From a micromechanical standpoint, the increase in cutting depth results in the activation of additional deformation mechanisms, including dislocation multiplication, phase transformation, and interfacial sliding. These mechanisms are enhanced by the mechanical resistance of CNTs, which promote strain hardening and localized yielding in the matrix. The evolution of shear strain with cutting depth reflects the material’s intrinsic ability to accommodate increased mechanical energy through distributed plastic flow, albeit constrained by the reinforcing phase. Collectively, the trends observed in Figure 8 and Figure 9 illustrate the complex interplay between cutting parameters and material behavior during nanomachining. High cutting speeds tend to suppress the diffusion of shear strain and promote localization due to thermal softening and rapid energy input. In contrast, increasing cutting depth expands the deformation zone, increases the interaction volume between tool and reinforcement, and leads to more distributed but still constrained shear strain. These findings underscore the necessity of tailoring cutting parameters to control strain localization, optimize material removal, and manage defect evolution in CNT-reinforced composites. The behavior of the shear band, including its angle, width, and internal strain distribution, offers valuable insights into the dynamic response of nanostructured materials under extreme mechanical and thermal conditions. The influence of CNTs on strain localization and deformation should therefore be considered in understanding the local stress distribution and plastic-flow behavior of the nanocomposite during cutting.

### 3.4. Surface Morphology and Microstructure Evolution

Figure 10 presents the relative atomic displacement of the workpiece atoms along the x, y, and z directions under different cutting speeds, providing critical insights into the influence of cutting speed on plastic deformation and chip formation. Panels (a1–d1) depict the atomic displacement in the cutting (x) direction. As cutting speed increases from 50 m/s to 200 m/s, a more pronounced backflow phenomenon is observed in the chip region. This backflow behavior is indicative of intensified plastic deformation driven by the enhanced cutting force and shear stress exerted by the tool. At higher cutting speeds, the driving force of the tool increases significantly, which not only facilitates material removal but also promotes the redirection of displaced atoms against the cutting direction due to localized stress wave reflection and recoil. This phenomenon signifies the formation of more dynamic deformation fields under high-speed conditions. Moreover, the narrowing of the primary shear zone and the intensification of chip backflow suggest that plastic deformation becomes increasingly localized as cutting speed rises. This is primarily attributed to the combined effects of elevated strain rates and temperature, which together enhance the plastic flowability of the material. The increased thermal energy under high-speed cutting facilitates bond weakening and atomic rearrangement in the shear zone, allowing for more efficient deformation and flow, but also inducing more concentrated stress and deformation zones. Panels (a2–d3) further reveal the formation and development of “surface bulges” on the machined workpiece surface. At lower cutting speeds, particularly 50 m/s, these bulges are more prominent, indicating an accumulation of plastically deformed material near the tool–workpiece interface. This accumulation arises from the insufficient flow rate of material in front of the tool, causing atoms to pile up and protrude along the surface. The restricted flowability is a direct consequence of lower thermal softening and reduced strain rate, resulting in broader but less intense plastic deformation zones. The role of carbon nanotubes (CNTs) becomes especially significant in these conditions. Due to their high Young’s modulus and exceptional tensile strength, CNTs effectively constrain the deformation of the surrounding matrix, impeding excessive flow and acting as nanoscale barriers to dislocation motion. This constraint increases the material’s resistance to plastic deformation, thereby exacerbating surface accumulation at lower speeds. Under high-speed cutting (e.g., 200 m/s), although local temperatures and interfacial stresses rise substantially—potentially leading to partial debonding or sliding at the CNT–matrix interface—CNTs continue to exert a crucial influence on deformation behavior. Their role transitions from a static mechanical constraint at low speed to a dynamic stress redistributor at high speed. By dispersing localized stresses and mitigating crack propagation, CNTs contribute to the stabilization of the shear zone and maintain surface integrity even under extreme conditions. This dual-function reinforcement illustrates the robustness of CNTs across a wide range of cutting parameters, underscoring their effectiveness in enhancing nanoscale machining stability.

Figure 11 provides a detailed examination of the relative atomic displacement patterns along the x, y, and z directions under varying cutting depths, offering further insight into the deformation behavior of CNT-reinforced metal composites. Panels (a1–d2) highlight the displacement evolution along the y-direction (perpendicular to cutting direction). At a shallow cutting depth of 5 Å, an “inclined collapse” phenomenon is observed in the chip region, suggesting that plastic deformation is highly localized and directional. The shallow engagement between the cutting tool and the material limits the extent of subsurface plasticity, resulting in a confined shear zone with minimal displacement diffusion into the surrounding material. As the cutting depth increases to 15 Å and 20 Å, the displacement range in the y-direction expands markedly. This indicates that deeper tool penetration leads to more extensive plastic flow, facilitated by the increased contact area and interaction time between the tool and the material. Notably, once the cutting depth exceeds 10 Å, the influence of CNTs on deformation becomes increasingly prominent. Due to their superior mechanical properties, CNTs enhance the composite’s resistance to shear and compressive forces, causing a redistribution of the strain field and altering the deformation mechanism from simple slip-dominated flow to one involving more complex interfacial sliding and constraint effects. At greater depths, the increased tool–CNT contact activates more resistance pathways, which not only strengthen the workpiece against deformation but also shift the plastic zone away from the immediate tool–matrix interface. This contributes to the observed increase in y-direction displacement range, as material flow must redistribute around more rigid inclusions. Panels (a3–d3) depict the atomic displacement behavior in the z-direction (depth direction). At a cutting depth of 20 Å, the emergence of pronounced “surface bulges” is evident in this direction. This is primarily attributed to the high-stress environment created by deep cutting, which leads to localized plastic flow resistance and stress accumulation near the surface. In these regions, the CNT–matrix interface may experience partial failure—such as debonding, sliding, or micro-delamination—under excessive interfacial shear stress. These micro-failures allow for sudden release of strain energy, manifesting as localized out-of-plane displacements, or surface bulging. The reinforcing function of CNTs in this context is twofold. On one hand, their high stiffness restricts matrix deformation, particularly along the z-direction, creating significant stress concentrations. On the other hand, when the stress exceeds the interfacial bonding threshold, CNTs may undergo relative sliding with respect to the surrounding matrix, altering the local strain distribution and flow dynamics. This behavior introduces additional complexity to the plastic flow process, as the interaction between tool, matrix, and reinforcement becomes highly nonlinear and strain-path-dependent. Such complex displacement patterns reveal that both cutting speed and depth substantially influence the plastic flow characteristics and surface morphology evolution during nanocutting. The relative displacement fields reflect the combined effects of thermal activation, mechanical loading, and interfacial phenomena, all modulated by the presence of CNTs. At lower cutting depths and speeds, CNTs primarily serve as mechanical constraints, whereas at higher parameters, their role shifts toward regulating stress distribution and stabilizing deformation through load redistribution and interfacial interaction.

### 3.5. Subsurface Defect Structure and Atomic Structure Evolution

Figure 12 illustrates the substantial influence of cutting speed on the formation, evolution, and spatial distribution of subsurface defects within the workpiece. A clear trend is observed: as the cutting speed increases, the thickness of the subsurface defect layer progressively decreases. This reduction is primarily attributed to the intensification of local strain rates and thermal effects induced by high-speed cutting. At elevated cutting speeds, the dynamic interaction between the tool and the workpiece leads to rapid material shearing, resulting in more concentrated plastic deformation confined to narrow zones near the cutting interface. The thermomechanical coupling effect becomes increasingly significant under high-speed conditions. As both temperature and stress increase locally, atomic mobility is enhanced, and the accumulation and growth of complex defect structures—such as extended dislocations or amorphous zones—are suppressed. Consequently, defect refinement is promoted, and the subsurface defect layer becomes thinner and more localized. In contrast, at lower cutting speeds, the tool maintains prolonged contact with the material. This extended interaction time leads to gradual stress accumulation and larger heat-affected zones, fostering the formation of more intricate defect structures. These include dislocation entanglements, micro-voids, and distorted atomic clusters. The longer exposure allows for stress redistribution and the emergence of complex defect morphologies that are distributed across a wider subsurface region. Furthermore, as the cutting distance increases from 150 Å to 200 Å, a noticeable expansion in the subsurface defect region is observed. This is due to the cumulative effect of continuous material removal and extended tool–workpiece interaction. The increasing cutting distance promotes sustained plastic deformation, intensifies local stress concentrations, and further activates atomic rearrangement processes. As a result, a broader variety of defects emerges, ranging from simple point defects and stacking faults to more refined structures such as atomic clusters and defect networks. These complex formations are characteristic of highly stressed and dynamically evolving subsurface regions in prolonged cutting regimes. The refinement of defect morphology under extended cutting also reflects the enhanced dislocation activity and the fragmentation of pre-existing defect structures, ultimately contributing to a more intricate and mechanically responsive subsurface zone.

Figure 13 explores the effect of cutting depth on the distribution and complexity of subsurface defects, revealing a non-monotonic trend in defect layer thickness. Specifically, the thickness initially decreases with increasing cutting depth, reaching a minimum at 10 Å, and then increases again, with the thickest defect layer occurring at a cutting depth of 20 Å. This variation highlights the critical role of tool penetration depth in regulating subsurface stress fields and defect nucleation. At a shallow cutting depth of 5 Å, the tool interacts primarily with the matrix material without engaging the embedded carbon nanotubes (CNTs). The deformation remains relatively mild, resulting in limited plastic strain and a moderately stable subsurface region. Defect structures in this stage are sparse and simple, mostly comprising minor dislocations and point defects induced by elastic and quasi-plastic deformation. When the cutting depth increases to 10 Å, the tool begins to interact with the outer periphery of the CNT-reinforced zone. Despite this contact, the interaction remains superficial, and the overall thermomechanical response is more controlled. As a result, the defect layer further thins, and the defect types become more uniform, indicating a transitional regime where the reinforcing effect of CNTs suppresses excessive deformation and defect accumulation. As the cutting depth continues to increase—especially at 15 Å—the contact length and area between the cutting tool and CNTs increase substantially. The CNTs, known for their exceptional stiffness and load-bearing capability, impose greater constraints on the surrounding matrix, altering the stress distribution and triggering more intense shear localization. This leads to an increase in defect complexity and the emergence of highly localized structures such as V-shaped dislocations, atomic disorder zones, stacking faults, and stacking fault tetrahedra (SFTs). The appearance of SFTs, in particular, suggests that the material is undergoing high-energy deformation events typically associated with severe lattice distortion and atomic slip along multiple planes. At a cutting depth of 20 Å, the tool penetrates even deeper into the CNT-reinforced region, resulting in maximum tool–CNT interaction and a pronounced concentration of mechanical stress in the subsurface. The intense constraint imposed by CNTs on the surrounding matrix generates severe lattice incompatibility, leading to stress-induced interfacial sliding or debonding. This, in turn, creates conditions conducive to vacancy generation and dislocation multiplication. The defect layer becomes the thickest at this stage, populated with high-density vacancy clusters, split dislocations, and interface-related defects. These features indicate that the material undergoes complex failure mechanisms, including void nucleation and local phase transformation, due to the overwhelming localized strain and stress concentrations introduced by deep tool penetration.

Overall, Figure 12 and Figure 13 collectively demonstrate how both cutting speed and cutting depth intricately influence the evolution of subsurface defects in CNT-reinforced metal matrices. High cutting speeds tend to confine defect development through enhanced plastic flow and thermal softening, while greater cutting depths increase the severity and diversity of defects due to intensified mechanical loading and matrix–CNT interactions. Understanding these interactions provides critical guidance for optimizing nanocutting parameters to minimize structural damage, preserve surface integrity, and enhance the performance of nanocomposite materials during ultra-precision machining.

Figure 14 reveals the trend of atomic structure evolution under different cutting speeds. As the cutting distance increases, the number of atoms classified as FCC gradually decreases, whereas the populations identified as HCP, BCC, and other/disordered local environments generally increase. The reduction in FCC-classified atoms becomes more pronounced at higher cutting speeds, indicating increasingly severe lattice distortion and local structural disorder. In the CNA, HCP-classified atoms in an FCC Ni matrix are primarily associated with local stacking-fault- or twin-related environments, whereas BCC-classified and other atoms may represent highly distorted local configurations rather than stable bulk phases. Therefore, these changes are interpreted as evidence of increasing local structural disorder and defect evolution rather than unequivocal bulk phase transformation. Specifically, at a cutting speed of 100 m/s, the rate of increase in HCP structure atoms is significantly higher, and at 200 m/s, the number of HCP atoms is about four times that at 100 m/s, indicating that lattice distortion and local transformations are exacerbated during high-speed cutting. Under high cutting speeds, the constraint effect of CNTs, as reinforcements, is significantly weakened due to the concentration of cutting forces, which results in an increased proportion of BCC and HCP structures. Although CNTs suppress dislocation diffusion and crack propagation to some extent, their interaction with the matrix becomes more complex under high-speed cutting, leading to a reduction in CNT reinforcement, which in turn promotes the formation of BCC and HCP lattices and a significant increase in amorphous atoms.

Figure 15 demonstrates the effect of cutting depth on atomic structure evolution. As shown in Figure 15, as the cutting depth increases, the number of FCC structures gradually decreases from an initial value of 683,565 to 649,716, while at smaller cutting depths (e.g., 5 Å), the FCC structure remains relatively intact. This phenomenon indicates that at smaller cutting depths, the stress is lower, and the material lattice undergoes fewer changes, leading to a more stable FCC structure. As the cutting depth increases, particularly at 20 Å, the reduction in FCC-classified atoms becomes more pronounced, while the number of atoms classified as other/disordered local environments increases to 125,030, representing a 1.14-fold increase compared with that at a cutting depth of 5 Å. Additionally, the number of HCP structure atoms increases significantly with cutting depth, especially at 15 Å and 20 Å, where it surpasses 8000. This increase in HCP-classified atoms indicates more extensive stacking-fault-related local environments and lattice distortion at larger cutting depths. This phenomenon is due to the increased contact area between the tool and the workpiece at larger cutting depths, which enhances the stress concentration effect in the cutting region. When the tool penetrates the CNT reinforcement area, the stress concentration in local regions becomes more prominent, exacerbating lattice transformation. Under these conditions, although the reinforcing effect of CNTs suppresses some dislocation diffusion, it is insufficient to completely prevent the formation of BCC and HCP structures. Instead, it intensifies the transformation of FCC structures into BCC and HCP structures and further promotes the generation of more amorphous structures and stacking faults in local areas.

### 3.6. Dislocation Evolution

Figure 16 shows the dislocation line distribution in the subsurface region of the workpiece at different cutting speeds. As observed, dislocations primarily consist of 1/6<112> (Shockley) dislocations during the cutting process. At lower cutting speeds (v = 50 m/s), shear stress and thermal effects are relatively minimal, restricting dislocation slip. As a result, dislocations accumulate mainly near the surface and subsurface regions, and their distribution remains confined. However, as the cutting speed increases to 100 m/s, the range of dislocation slip expands, with dislocation lines extending deeper into the subsurface region. The strengthening effect of CNTs is particularly evident in this process. Due to their high strength and rigidity, CNTs somewhat impede dislocation motion, slowing down dislocation propagation. At a cutting speed of 200 m/s, CNTs exhibit a significant regulatory effect. The interface constraint mechanism of CNTs effectively prevents large-scale dislocation slip, causing dislocations to form and halt in localized regions, which suppresses dislocation propagation. However, at high cutting speeds, the combined effects of high temperature and stress can cause slip at the CNT-matrix interface, leading to more localized dislocation distributions and an increased occurrence of dislocation annihilation. This suggests that while CNTs can effectively hinder dislocation slip under certain conditions, their reinforcing effect is limited by temperature and stress in extreme cutting environments.

Figure 17 illustrates how the densities of different dislocation types change with cutting distance at various cutting speeds. The results indicate that as cutting distance increases, the densities of all dislocation types tend to rise. At lower cutting speeds (v = 50 m/s), the density of 1/6<110> (Stair-rod) dislocations significantly increases, indicating a strong accumulation of dislocations. Similarly, the density of 1/6<112> (Shockley) dislocations also increases. This behavior is attributed to the notable constraint effect of CNTs in the cutting region during low-speed cutting. The high strength and elasticity of CNTs limit local deformation, causing shear stress to concentrate in specific areas, thus promoting dislocation formation and accumulation. In contrast, at higher cutting speeds (v = 200 m/s), the densities of all dislocation types remain relatively low, with the density of 1/6<110> (Stair-rod) dislocations showing almost no noticeable increase. At v = 200 m/s, the density of 1/6<110> dislocations is only 1/2.4 of that at v = 50 m/s, indicating that the high thermal stability and rigidity of CNTs at high cutting speeds help maintain the material’s lattice integrity, thereby suppressing dislocation formation and propagation. This phenomenon also shows that under high-speed cutting conditions, CNTs not only limit dislocation propagation but also assist in slowing further dislocation accumulation by promoting localized temperature increases and plastic flow. In particular, at a cutting speed of 100 m/s, 1/6<112> (Shockley) dislocations show an extremely high sensitivity, with their peak density reaching 3.1 times that at 200 m/s, suggesting that CNTs have a more limited effect on dislocation suppression at this cutting speed. During low-speed cutting, the high elasticity and strength of CNTs effectively promote dislocation generation and accumulation under localized shear stress, making 1/6<112> (Shockley) dislocations the preferentially activated slip system. This suggests that the reinforcing effect of CNTs is closely related to cutting speed, and at lower speeds, CNTs significantly promote dislocation formation through localized stress concentration.

Figure 18 shows the distribution of dislocation lines at different cutting depths. As cutting depth increases, dislocation distribution progressively extends from the surface deeper into the substrate, indicating that cutting depth has an increasing influence on material plastic deformation. At shallow cutting depths (5 Å), dislocation lines are primarily concentrated on the surface, predominantly consisting of 1/6<112> (Shockley) dislocations. This occurs because, at shallow depths, material deformation is confined to the surface layer, resulting in simpler dislocation slip paths with dislocations concentrated near the surface. As cutting depth increases, dislocation lines extend further into the substrate. At a cutting depth of 15 Å, the tool reaches the CNT region, and the high strength and modulus of CNTs begin to significantly hinder dislocation slip. This hindrance alters the dislocation distribution pattern and induces a noticeable increase in dislocation entanglement beneath the tool contact area. The dislocation entanglement not only restricts material plastic flow but also exacerbates the annihilation of 1/3<100> (Hirth) dislocations, making plastic flow in stress concentration areas more complex. When cutting depth increases to 20 Å, CNTs continue to strengthen the material in localized regions. Their high strength and rigidity increase the local resistance to deformation, while the restricted plastic flow is accompanied by enhanced stress concentration in localized regions.

Figure 19 illustrates the variation in dislocation densities with cutting distance for different dislocation types at various cutting depths. The data show that at shallower cutting depths (e.g., 5 Å), dislocation densities are relatively low, and the reinforcing effect of CNTs is not fully realized. However, as cutting depth increases to 10 Å and 15 Å, dislocation densities rise significantly, particularly the densities of 1/6<112> (Shockley) and 1/2<110> (Perfect) dislocations. During this process, the fluctuation of dislocation density with cutting distance also increases significantly. This behavior suggests that deeper cutting depths promote material plastic deformation, with CNTs effectively altering dislocation slip paths, hindering dislocation motion, and promoting more dislocation interactions. This results in a notable increase in dislocation density and its fluctuations. When cutting depth increases to 20 Å, all dislocation types show a significant increase in density and exhibit stronger fluctuations. In particular, 1/6<110> (Stair-rod) and 1/6<112> (Shockley) dislocations are particularly sensitive to changes in cutting depth. At this depth, their densities increase by 8.4 times and 5.2 times, respectively. This indicates that, at deeper cutting depths, internal material stress and plastic flow reach higher levels, increasing stress concentration and leading to more complex dislocation generation and annihilation. Furthermore, at greater cutting depths, CNTs have a more pronounced effect on hindering dislocation slip and entanglement. The high strength and modulus of CNTs significantly increase the deformation resistance during cutting, further promoting dislocation density and making deformation in the cutting zone more complex. CNTs not only alter the dislocation distribution pattern but also exacerbate dislocation entanglement and hinder slip, resulting in a substantial increase in dislocation density.

### 3.7. Discussion

The nanocutting behavior of carbon nanotube (CNT)-reinforced Ni-based composites presents a complex interplay between mechanical, thermal, and interfacial phenomena at the atomic scale. Through molecular dynamics simulations, this study has revealed how cutting parameters, including cutting speed and depth, govern the dynamic interactions and deformation mechanisms in such nanocomposites. The findings not only offer atomic-scale insights into material behavior during high-precision machining but also highlight the pivotal role of CNTs in regulating dislocation evolution, subsurface defect generation [46,47], and phase transformation. Previous atomistic studies have examined nanocutting behavior in Ni-containing and other metallic systems, including NiTi alloys [26], high-entropy alloys containing Ni [48], and Ni-based alloys [35], demonstrating the important influence of cutting parameters on local stress, deformation, and subsurface structural evolution. Studies on CNT-reinforced metallic composites have also shown that CNTs can modify local load transfer and deformation behavior [46,47]. Compared with these studies, the present work focuses on the coupled evolution of cutting force, temperature, stress–strain response, local atomic structure, and dislocation behavior in a CNT–Ni system under systematically varied cutting speed and cutting depth. Qualitatively, the decrease in cutting force accompanied by an increased thermal response observed in the present study is consistent with previous MD results for NiTi nanocutting, where elevated cutting temperature was associated with reduced cutting force and residual stress [25]. In addition, previous simulations of FeCoNiCrAl_0.6_ alloys have shown that nanoscale cutting parameters strongly influence cutting force, shear deformation, dislocation activity, chip characteristics, and subsurface damage [23], which is broadly consistent with the parameter-dependent trends observed here. Direct comparison with experimental nanomachining results remains limited because the present study focuses on atomistic-scale trends under idealized MD conditions. The results further indicate that the effects of cutting conditions and CNT–matrix interactions should be considered separately when interpreting the observed deformation mechanisms.

One of the most significant findings is the dual role of cutting speed in modulating both mechanical resistance and thermal softening. As the cutting speed increased from 50 m/s to 200 m/s, a notable reduction in cutting forces was observed, accompanied by a rise in cutting temperature. The thermal softening effect facilitated plastic deformation while simultaneously reducing the extent of subsurface damage and defect propagation [49]. This aligns with the observed decrease in the thickness of the subsurface defect layer and the simplification of defect types under high-speed conditions. Interestingly, although high cutting speeds promote thermal activation and amorphization, they also localize deformation and confine plastic flow within narrower shear bands. This localization reduces overall stress diffusion and helps maintain surface integrity, particularly when CNTs act as rigid reinforcements that modulate strain localization. In contrast, increasing cutting depth resulted in a non-linear influence on both cutting force and structural evolution [50]. Initially, deeper cuts intensified force fluctuations due to increased contact between the tool and CNTs. However, beyond a critical depth, the system stabilized, with smoother stress distribution and a transition toward more uniform plastic flow. This stabilization is attributed to the widening of the shear zone, which provides a broader energy dissipation path. Nevertheless, deeper cuts also led to more pronounced structural changes, including higher densities of dislocations, extensive stacking faults [42], and an increased population of atoms with disordered local environments. The number of FCC-classified atoms decreased markedly, while HCP- and BCC-classified populations increased, indicating severe lattice distortion and defect-associated structural rearrangement. These changes suggest that deeper cutting promotes the loss of local FCC ordering and increases structural disorder in the highly deformed region. These observations primarily reflect the overall thermomechanical response of the system to changes in cutting speed and cutting depth.

Within the present CNT–Ni configuration, pronounced plastic-flow localization and changes in dislocation behavior are observed near the CNT–matrix region as the cutting conditions vary. The high stiffness of CNTs and the local CNT–matrix interaction may contribute to the observed constraint of plastic flow and changes in dislocation pathways. At low cutting speeds, relatively high densities of 1/6<110> stair-rod and 1/6<112> Shockley dislocations are observed, which is associated with localized stress concentration in the CNT-reinforced region [51,52,53]. At higher cutting speeds, the rapid thermal response and localized deformation are accompanied by more complex changes in the dislocation network. With increasing cutting depth, stronger tool–CNT–matrix interactions are accompanied by increased stress localization and more complex dislocation structures. However, because a pure Ni reference system is not included in the present study, the specific contribution of CNT reinforcement cannot be quantitatively separated from the effects of cutting speed and cutting depth. The atomic displacement analysis further supports the conclusion that CNTs significantly modulate deformation behavior [54,55]. At lower cutting speeds, pronounced surface bulges and backflow phenomena were observed. In contrast, these features became less pronounced at higher cutting speeds, accompanied by changes in chip morphology and more localized deformation. Along the z-axis, surface protrusions at higher cutting depths were linked to CNT–matrix interfacial failure, underscoring the importance of maintaining strong interfacial adhesion during deep cuts to ensure machining stability [56]. Additionally, the evolution of atomic structures under different cutting conditions revealed key trends in phase transformation. High cutting speeds and deep cuts both contributed to a reduction in FCC atomic populations, with a corresponding increase in HCP, BCC, and amorphous structures [57,58]. These transitions are consistent with the thermally activated and stress-driven lattice distortions encountered during extreme nanocutting processes. Interestingly, while CNTs suppressed certain structural transformations at moderate cutting depths, their influence was less effective under extreme stress or high-temperature conditions, where interfacial sliding and debonding became more prevalent. Dislocation analysis further reveals that cutting speed and cutting depth produce distinct effects on dislocation evolution [59]. The dislocation density does not vary monotonically with cutting speed. At 50 m/s, pronounced accumulation of 1/6<110> (Stair-rod) dislocations is observed, whereas their density at 200 m/s is approximately 1/2.4 of that at 50 m/s. The 1/6<112> (Shockley) dislocations also exhibit a pronounced peak at 100 m/s, indicating a complex speed-dependent response. In contrast, increasing cutting depth generally leads to higher dislocation densities and stronger fluctuations. At a cutting depth of 20 Å, the densities of 1/6<110> and 1/6<112> dislocations increase by approximately 8.4 and 5.2 times, respectively, compared with the shallow-cutting condition. These results indicate that the effects of cutting speed and cutting depth on dislocation evolution should be considered separately. This indicates a transition from simple slip systems to complex dislocation networks and suggests that under extreme conditions, dislocation multiplication is no longer efficiently suppressed by CNTs.

Taken together, the present results show that cutting speed and cutting depth govern the overall thermomechanical and defect responses of the CNT–Ni system, while the presence of the CNT is associated with additional localization of stress, deformation, and dislocation activity in the surrounding matrix. These effects should be distinguished when interpreting the observed mechanisms, particularly because the present simulation does not include a pure Ni reference system. The combined analysis of atomic displacement, local atomic structure, and dislocation evolution therefore provides further insight into the response of the selected CNT–Ni configuration under different nanocutting conditions. Overall, the present results provide comparative atomistic trends regarding the effects of cutting speed and cutting depth on the deformation behavior of the CNT–Ni system. These trends may provide a useful reference for subsequent process studies; however, direct extrapolation to practical machining conditions should be made with caution because of the inherent spatial and temporal scale limitations of MD simulations. It should also be noted that the present study does not include a pure Ni reference model; therefore, the observed trends primarily characterize the response of the selected CNT–Ni system, and a direct quantitative assessment of the CNT reinforcement effect requires future comparison with pure Ni under identical cutting conditions. Because only a single 60 Å-long armchair (4,4) CNT with a fixed embedding configuration was considered, the present conclusions should be interpreted within this specific microstructural configuration. The effects of CNT chirality, orientation, and volume fraction require further investigation. Future research should further investigate the effects of CNT volume fraction, orientation, and surface modification on nanocutting performance. In addition, a direct comparison between CNT–Ni composites and pure Ni under identical cutting conditions will be conducted to quantitatively clarify the specific reinforcement effect of CNTs.

## 4. Conclusions

In this study, CNT-Ni composite materials were investigated to analyze their dynamic interactions and deformation mechanisms during the nanoscale cutting process. The key conclusions are as follows:

(1) Increasing cutting speed leads to pronounced changes in the mechanical, thermal, and structural responses of the CNT–Ni system. As the cutting speed increases from 50 m/s to 200 m/s, the average tangential cutting force decreases by approximately 16.5%, while the cutting temperature increases by 26.7% at a cutting distance of 200 Å. The evolution of dislocation density shows a non-monotonic dependence on cutting speed. Pronounced accumulation of 1/6<110> (Stair-rod) dislocations is observed at 50 m/s, whereas their density at 200 m/s is approximately 1/2.4 of that at 50 m/s. Meanwhile, the variation in local atomic environments indicates increased lattice distortion and structural disorder under high-speed cutting conditions.

(2) Increasing cutting depth produces stronger mechanical interaction between the tool and workpiece and generally leads to higher cutting forces, enhanced stress localization, and more extensive subsurface deformation. At a cutting depth of 20 Å, the tangential cutting force reaches approximately 1.84 times that at 5 Å, accompanied by increased structural disorder and more complex dislocation activity. In particular, the densities of 1/6<110> (Stair-rod) and 1/6<112> (Shockley) dislocations increase by approximately 8.4 and 5.2 times, respectively, compared with the shallow-cutting condition. These results indicate that deeper cutting promotes more intensive local deformation and defect evolution.

(3) Within the present CNT–Ni configuration, pronounced changes in plastic flow, local stress distribution, and dislocation behavior are observed near the CNT–matrix region as the cutting conditions vary. The local CNT–matrix interaction may contribute to the constraint of plastic flow and changes in dislocation pathways, while deeper cutting conditions further intensify the interaction among the tool, CNT, and Ni matrix. However, because a pure Ni reference system is not included in the present study, the specific contribution of CNT reinforcement cannot be quantitatively separated from the effects of cutting speed and cutting depth.

## Figures and Tables

**Figure 1:**
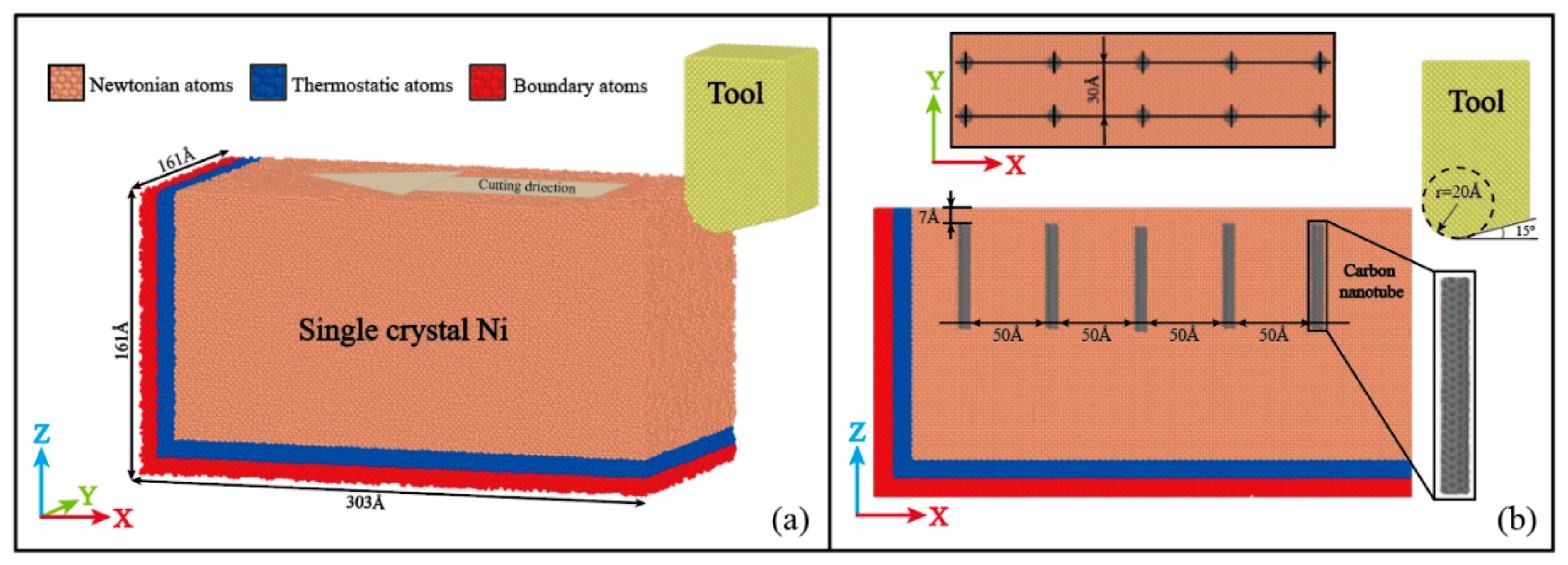
Illustration of the CNT–Ni composite nanocutting MD simulation model: (**a**) three-dimensional view; (**b**) cross-sectional view.

**Figure 2:**
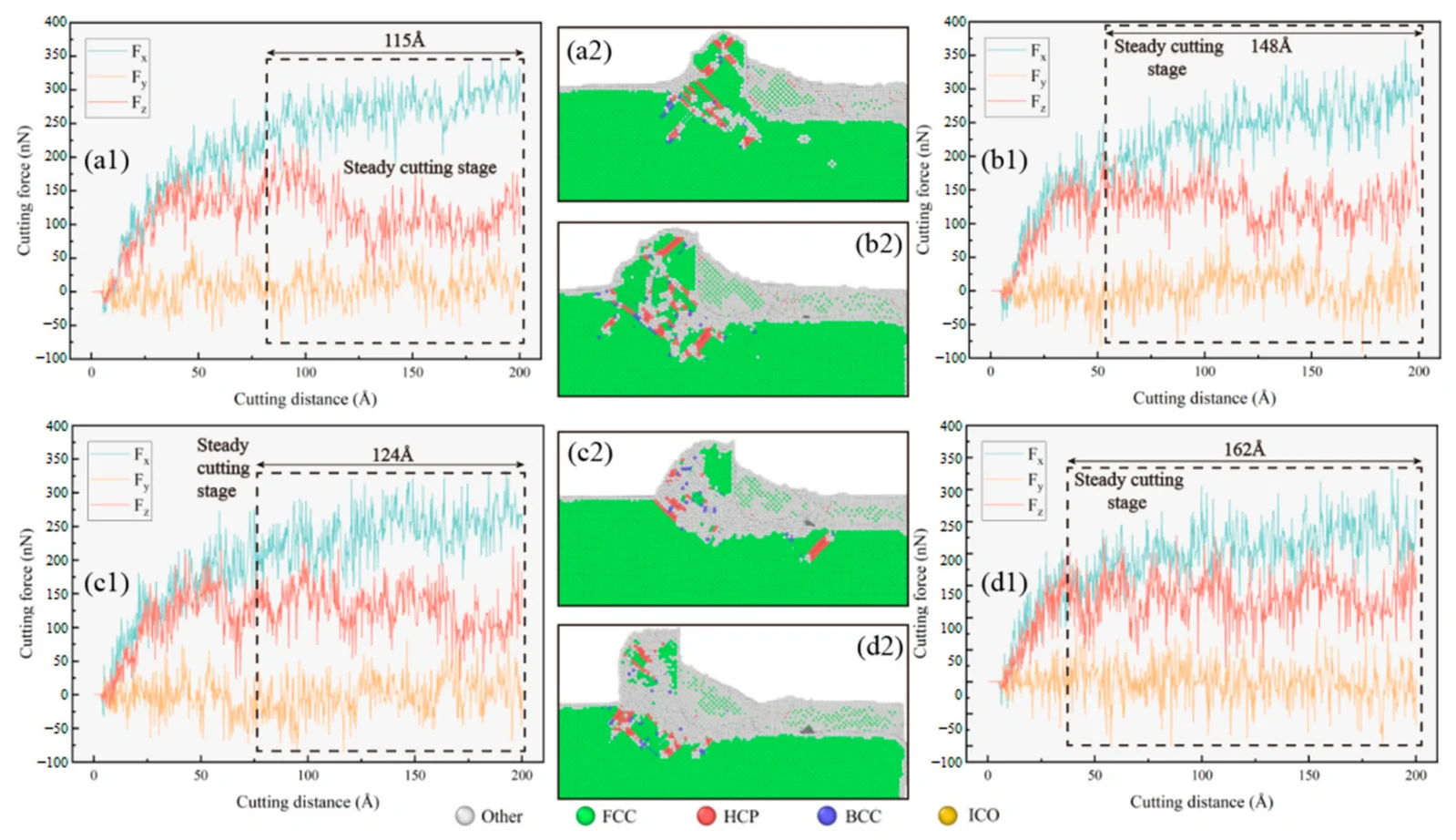
Cutting force fluctuations and defect distribution during the stable cutting phase at different cutting speeds. (**a1**,**a2**) v = 50 m/s; (**b1**,**b2**) v = 100 m/s; (**c1**,**c2**) v = 150 m/s; (**d1**,**d2**) v = 200 m/s.

**Figure 3:**
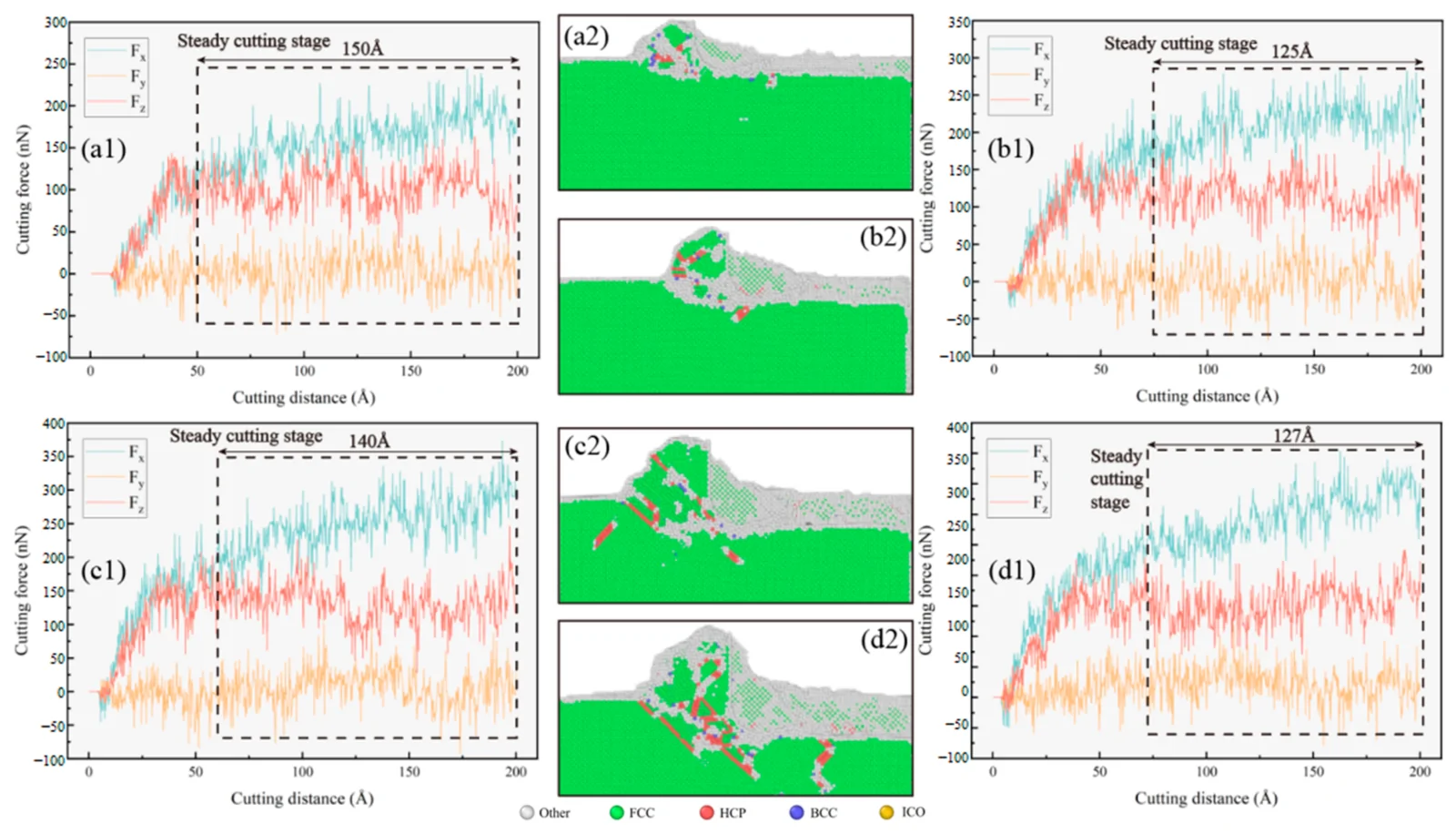
Cutting force fluctuations and defect distribution during stable cutting phase at different cutting depths. (**a1**,**a2**) ap = 5 Å; (**b1**,**b2**) ap = 10 Å; (**c1**,**c2**) ap = 15 Å; (**d1**,**d2**) ap = 20 Å.

**Figure 4:**
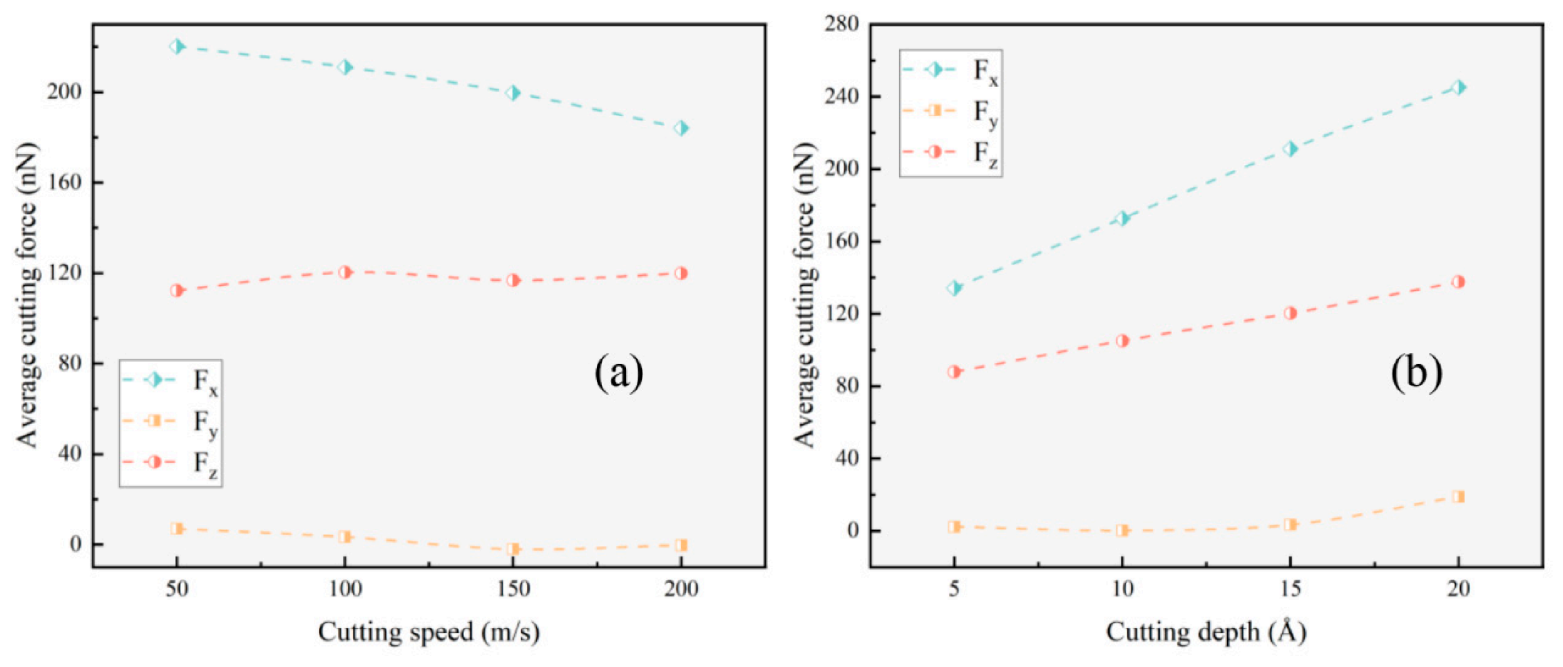
Evolution of average cutting force under different cutting parameters. (**a**) Different cutting speeds; (**b**) different cutting depths.

**Figure 5:**
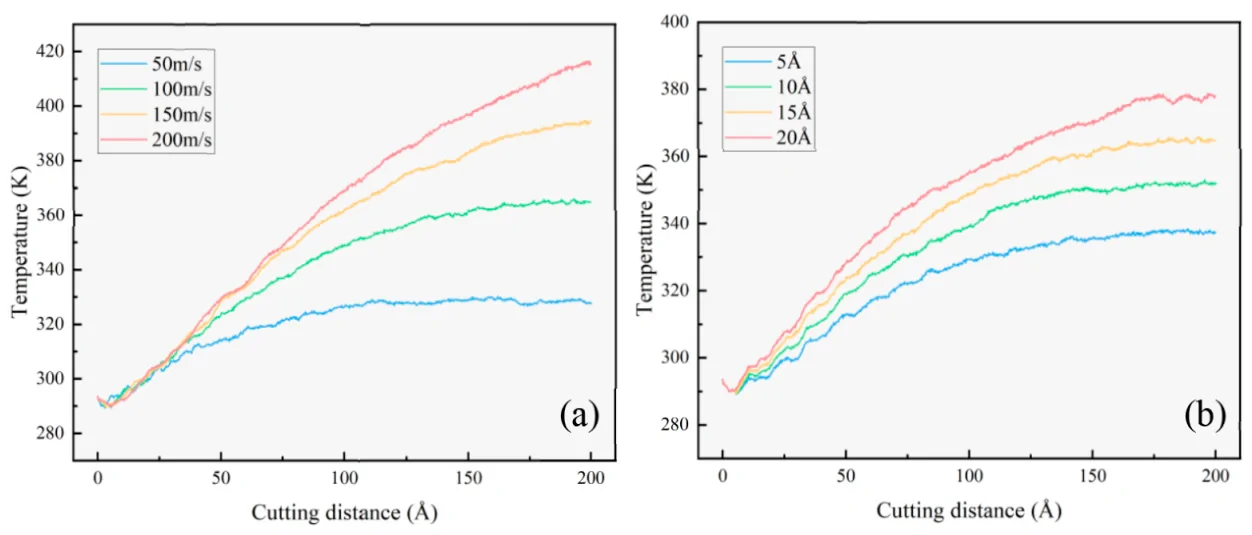
Evolution of cutting temperature with cutting distance under different cutting parameters. (**a**) Effect of cutting speed; (**b**) effect of cutting depth.

**Figure 6:**
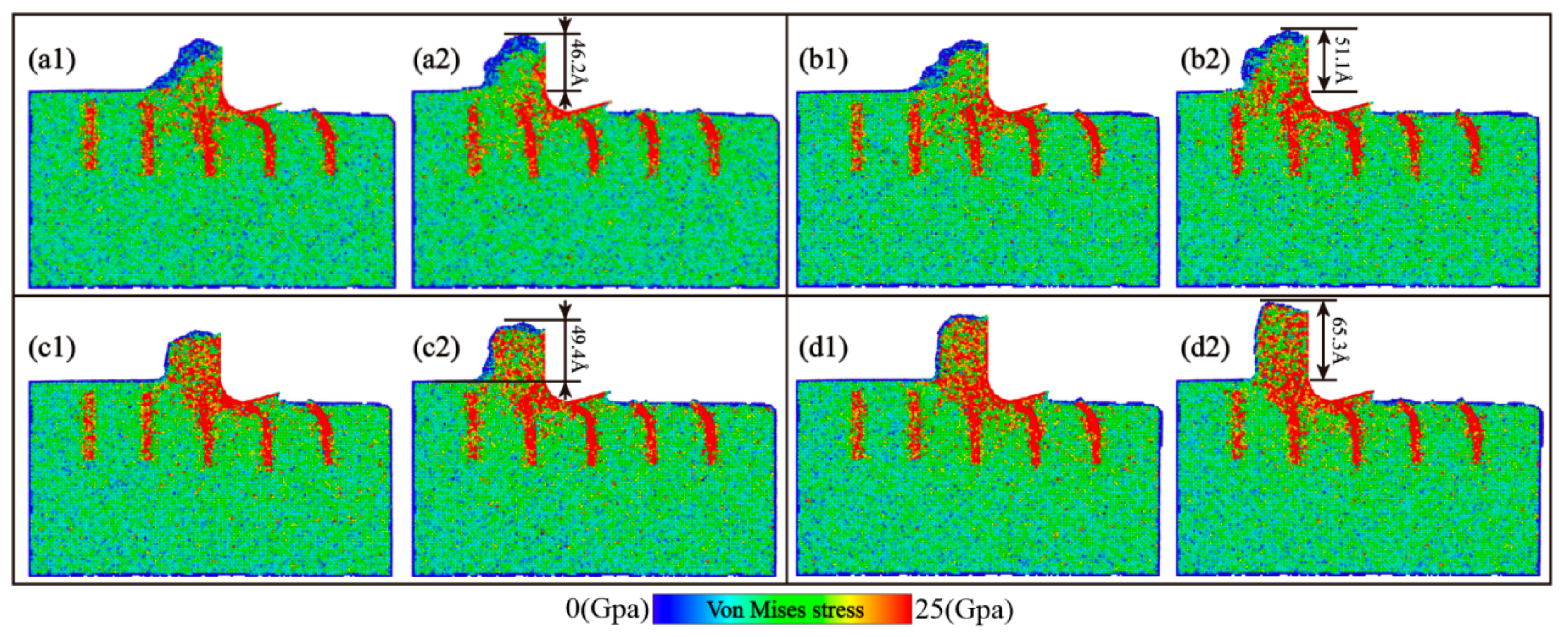
von Mises stress distribution at different cutting speeds. (**a1**) v = 50 m/s, l = 150 Å; (**a2**) v = 50 m/s, l = 200 Å; (**b1**) v = 100 m/s, l = 150 Å; (**b2**) v = 100 m/s, l = 200 Å; (**c1**) v = 150 m/s, l = 150 Å; (**c2**) v = 150 m/s, l = 200 Å; (**d1**) v = 200 m/s, l = 150 Å; (**d2**) v = 200 m/s, l = 200 Å.

**Figure 7:**
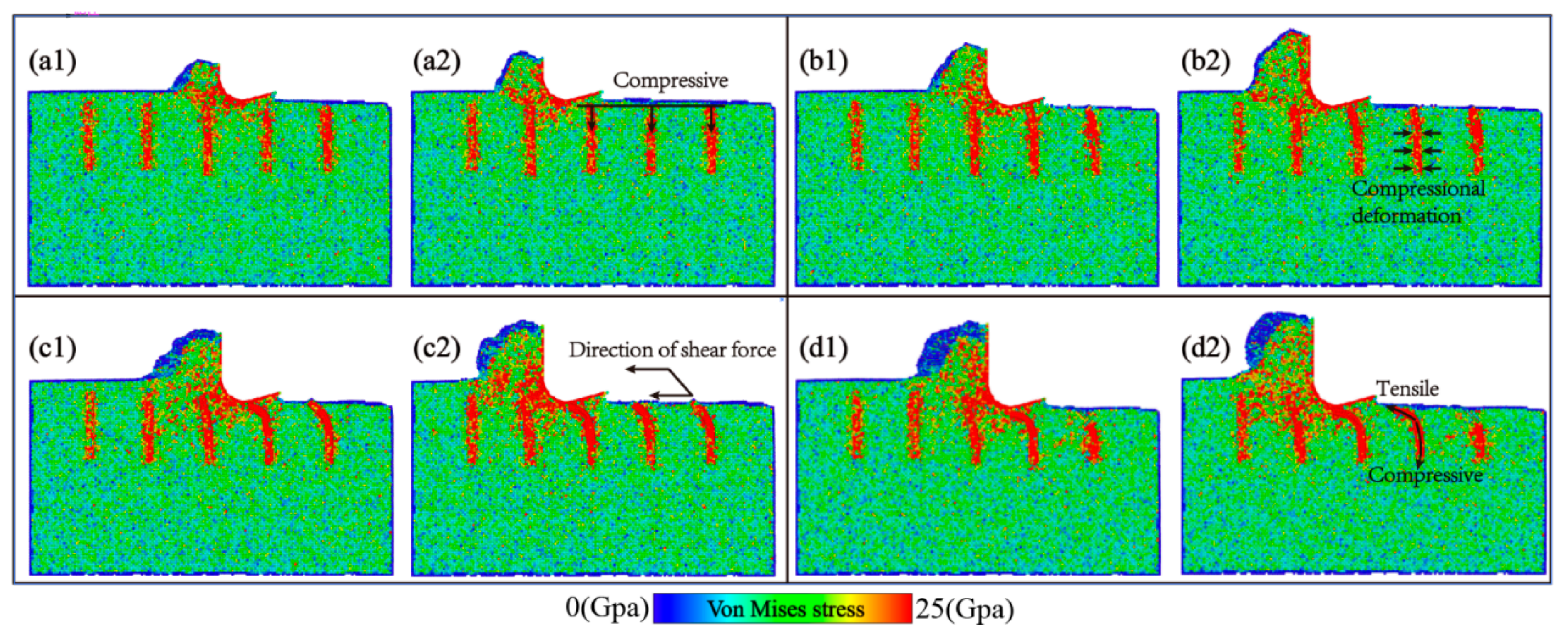
von Mises stress distribution at different cutting depths. (**a1**) ap = 5 Å, l = 150 Å; (**a2**) ap = 5 Å, l = 200 Å; (**b1**) ap = 10 Å, l = 150 Å; (**b2**) ap = 10 Å, l = 200 Å; (**c1**) ap = 15 Å, l = 150 Å; (**c2**) ap = 15 Å, l = 200 Å; (**d1**) ap = 20 Å, l = 150 Å; (**d2**) ap = 20 Å, l = 200 Å.

**Figure 8:**
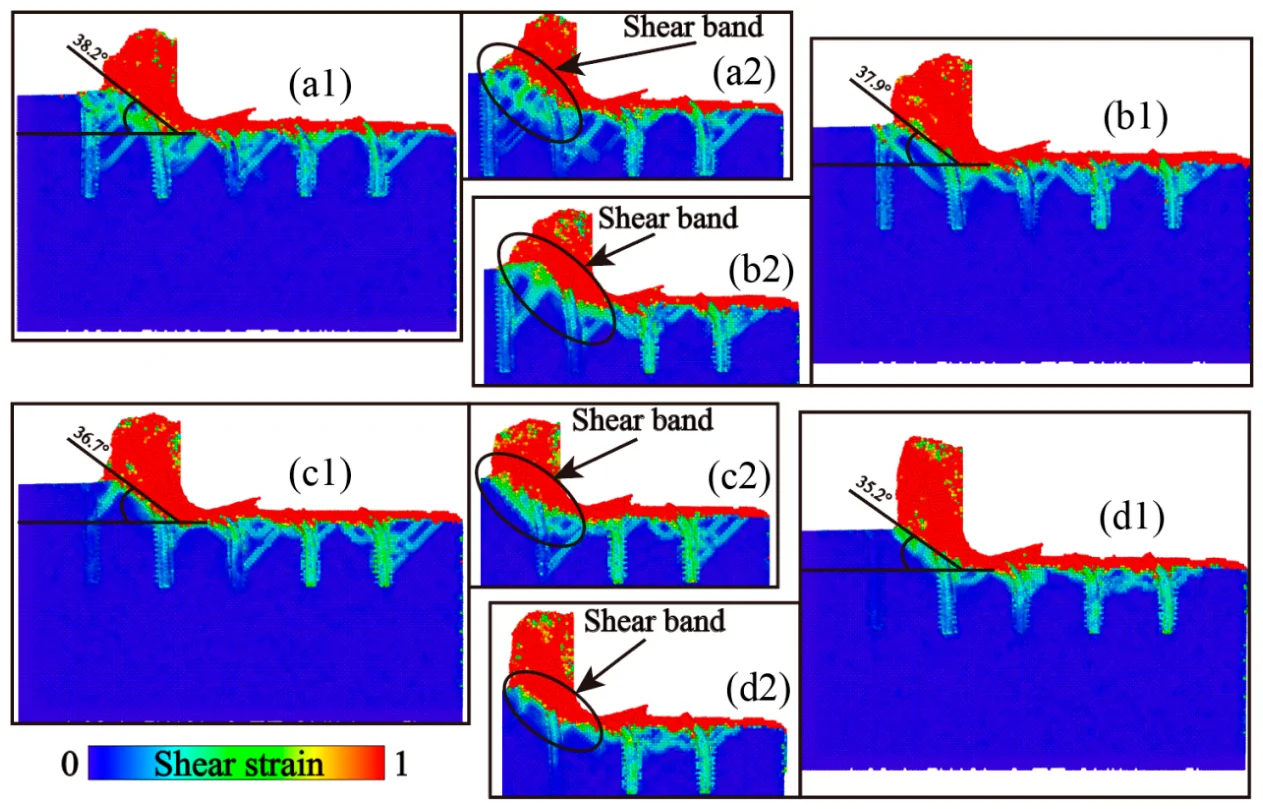
Shear strain distribution at different cutting speeds. (**a1**) v = 50 m/s, l = 200. Å; (**a2**) v = 50 m/s, l = 150 Å; (**b1**) v = 100 m/s, l = 200 Å; (**b2**) v = 100 m/s, l = 150 Å; (**c1**) v = 150 m/s, l = 200 Å; (**c2**) v = 150 m/s, l = 150 Å; (**d1**) v = 200 m/s, l = 200 Å; (**d2**) v = 200 m/s, l = 150 Å.

**Figure 9:**
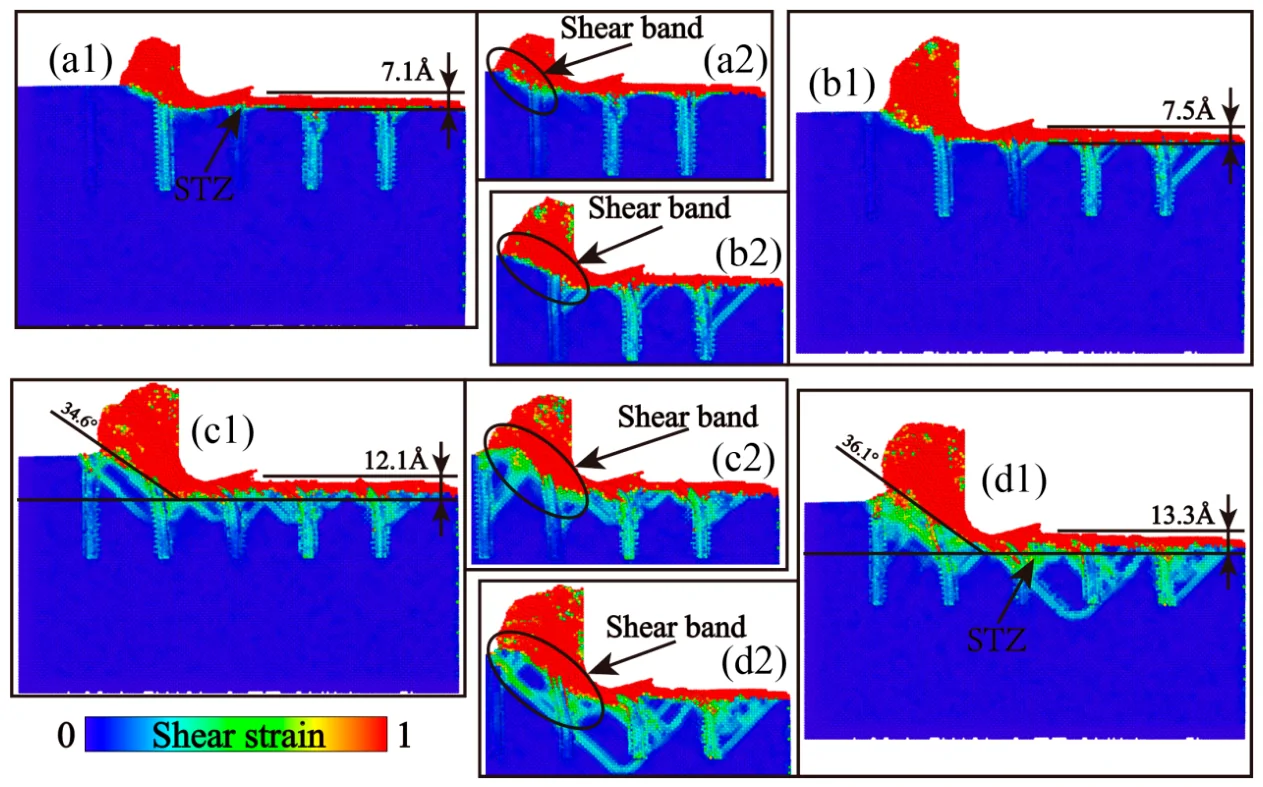
Shear strain distribution at different cutting depths. (**a1**) ap = 5 Å, l = 200 Å; (**a2**) ap = 5 Å, l = 150 Å; (**b1**) ap = 10 Å, l = 200 Å; (**b2**) ap = 10 Å, l = 150 Å; (**c1**) ap = 15 Å, l = 200 Å; (**c2**) ap = 15 Å, l = 150 Å; (**d1**) ap = 20 Å, l = 200 Å; (**d2**) ap = 20 Å, l = 150 Å.

**Figure 10:**
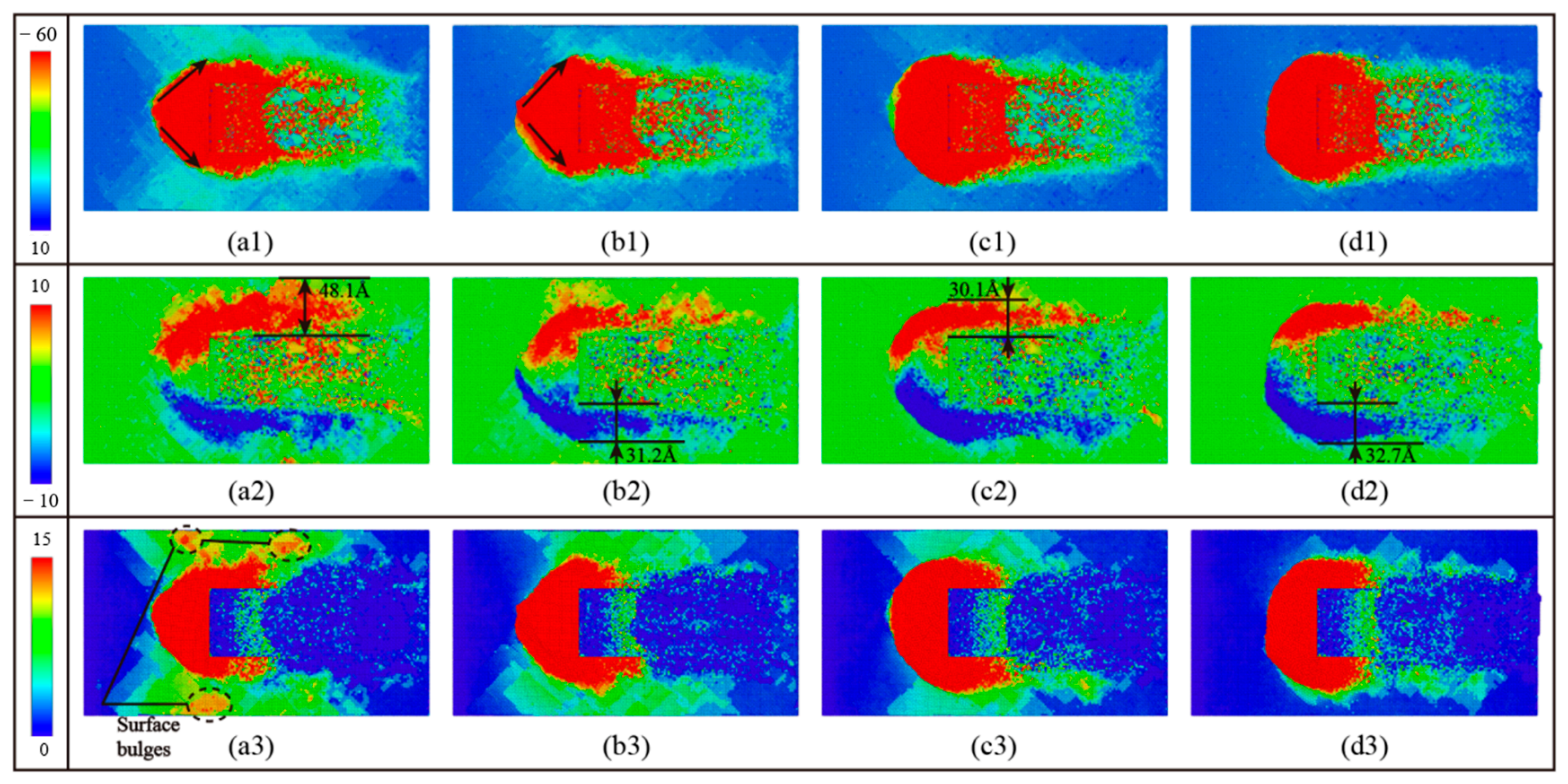
Relative atomic displacement of the workpiece in the x, y, and z directions under different cutting speeds. x-direction: (**a1**) 50 m/s, (**b1**) 100 m/s, (**c1**) 150 m/s, (**d1**) 200 m/s; y-direction: (**a2**) 50 m/s, (**b2**) 100 m/s, (**c2**) 150 m/s, (**d2**) 200 m/s; z-direction: (**a3**) 50 m/s, (**b3**) 100 m/s, (**c3**) 150 m/s, (**d3**) 200 m/s.

**Figure 11:**
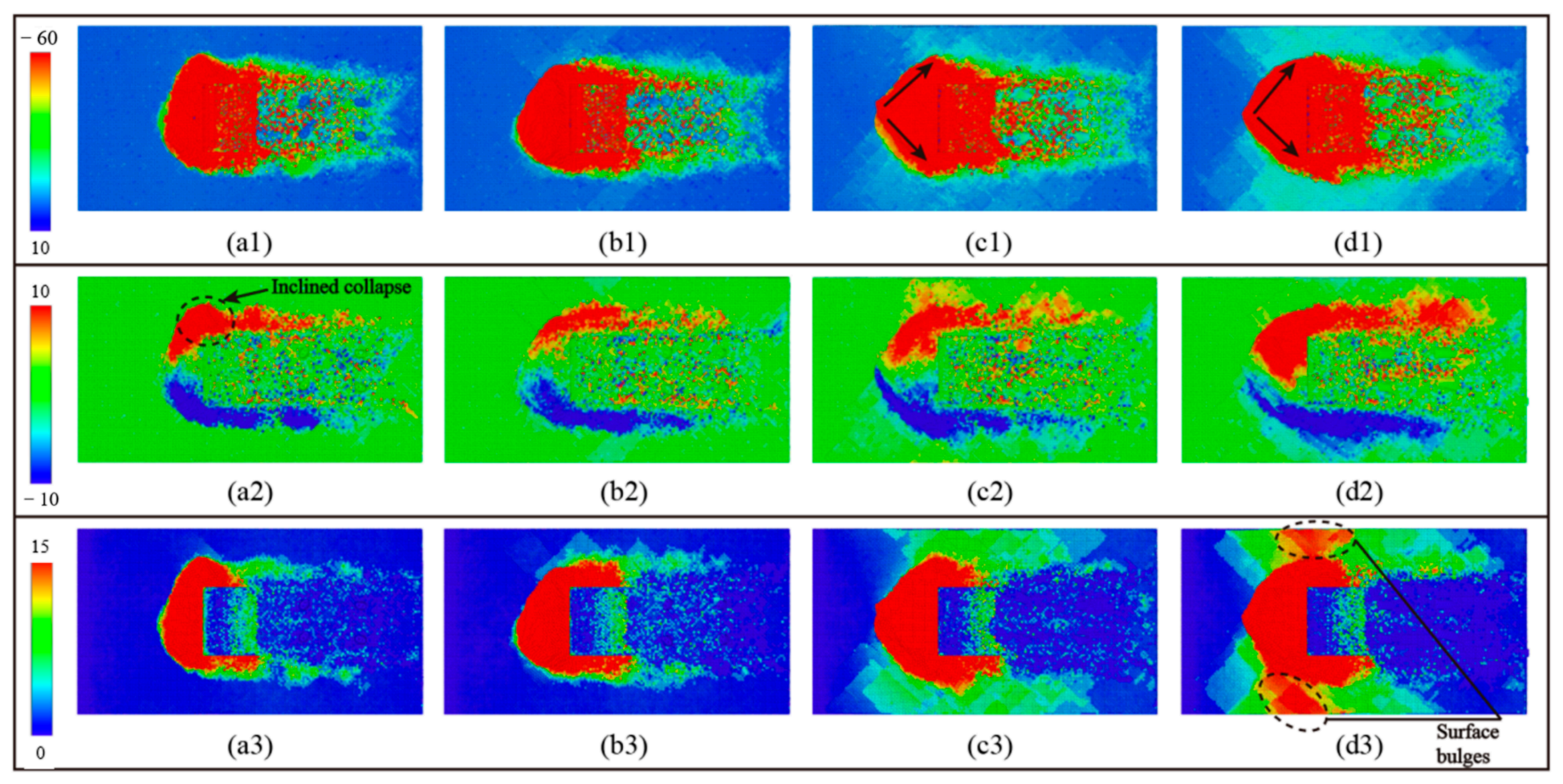
Relative atomic displacement of the workpiece in the x, y, and z directions under different cutting depths. x-direction: (**a1**) 5 Å, (**b1**) 10 Å, (**c1**) 15 Å, (**d1**) 20 Å; y-direction: (**a2**) 5 Å, (**b2**) 10 Å, (**c2**) 15 Å, (**d2**) 20 Å; z-direction: (**a3**) 5 Å, (**b3**) 10 Å, (**c3**) 15 Å, (**d3**) 20 Å.

**Figure 12:**
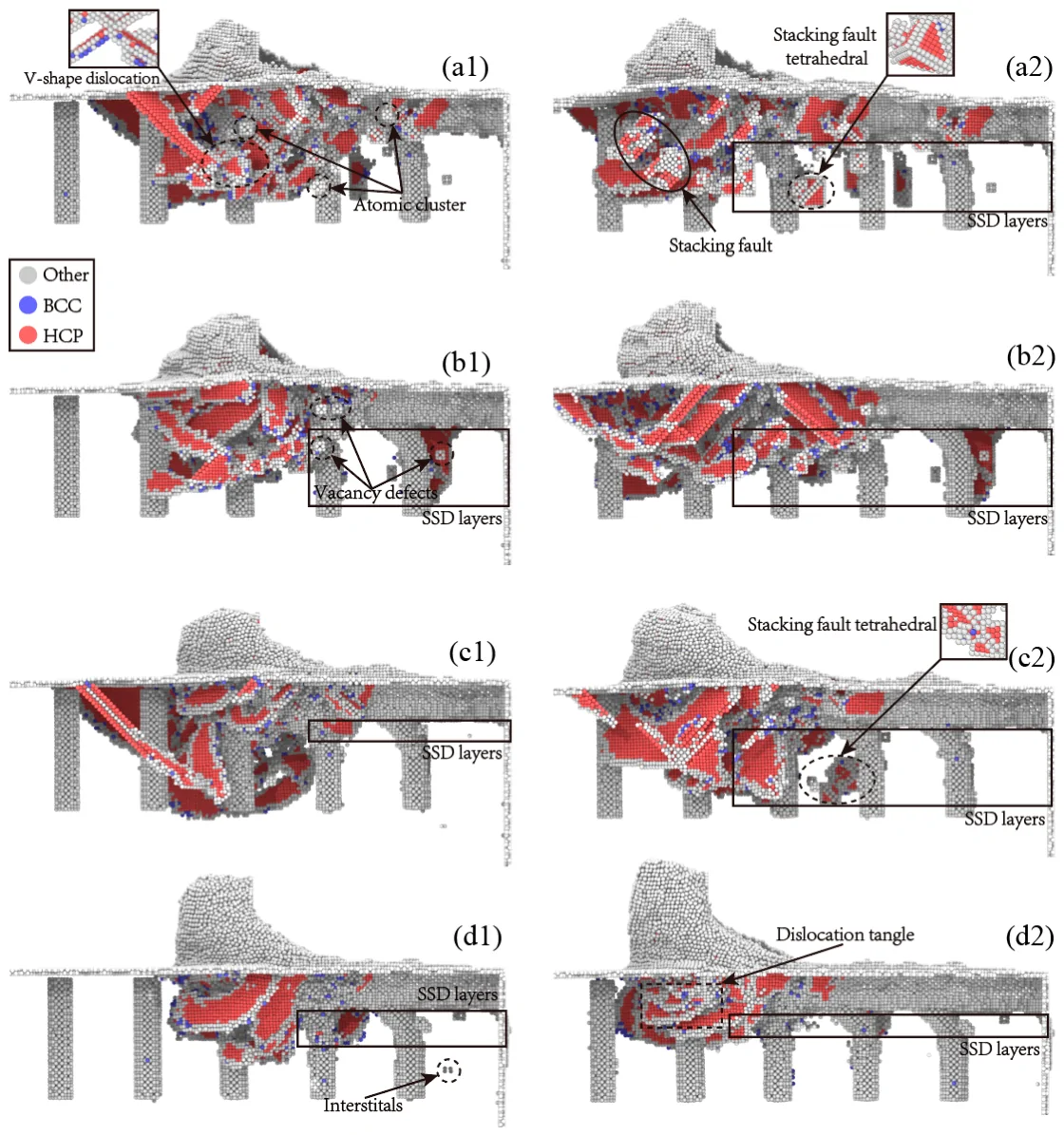
Subsurface defect structure distribution at different cutting speeds. (**a1**) v = 50 m/s, l = 150 Å; (**a2**) v = 50 m/s, l = 200 Å; (**b1**) v = 100 m/s, l = 150 Å; (**b2**) v = 100 m/s, l = 200 Å; (**c1**) v = 150 m/s, l = 150 Å; (**c2**) v = 150 m/s, l = 200 Å; (**d1**) v = 200 m/s, l = 150 Å; (**d2**) v = 200 m/s, l = 200 Å.

**Figure 13:**
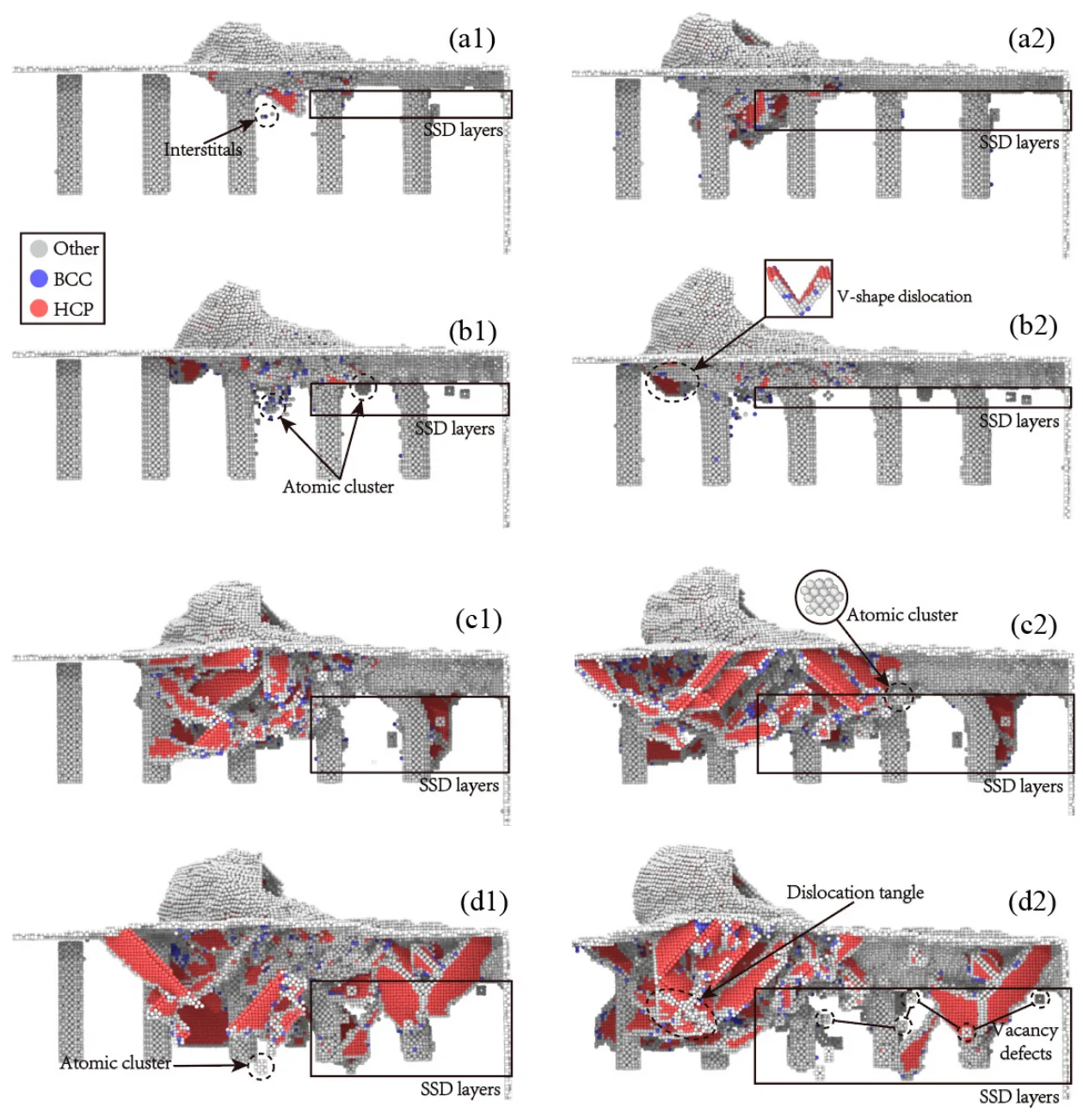
Subsurface defect structure distribution at different cutting depths. (**a1**) ap = 5 Å, l = 150 Å; (**a2**) ap = 5 Å, l = 200 Å; (**b1**) ap = 10 Å, l = 150 Å; (**b2**) ap = 10 Å, l = 200 Å; (**c1**) ap = 15 Å, l = 150 Å; (**c2**) ap = 15 Å, l = 200 Å; (**d1**) ap = 20 Å, l = 150 Å; (**d2**) ap = 20 Å, l = 200 Å.

**Figure 14:**
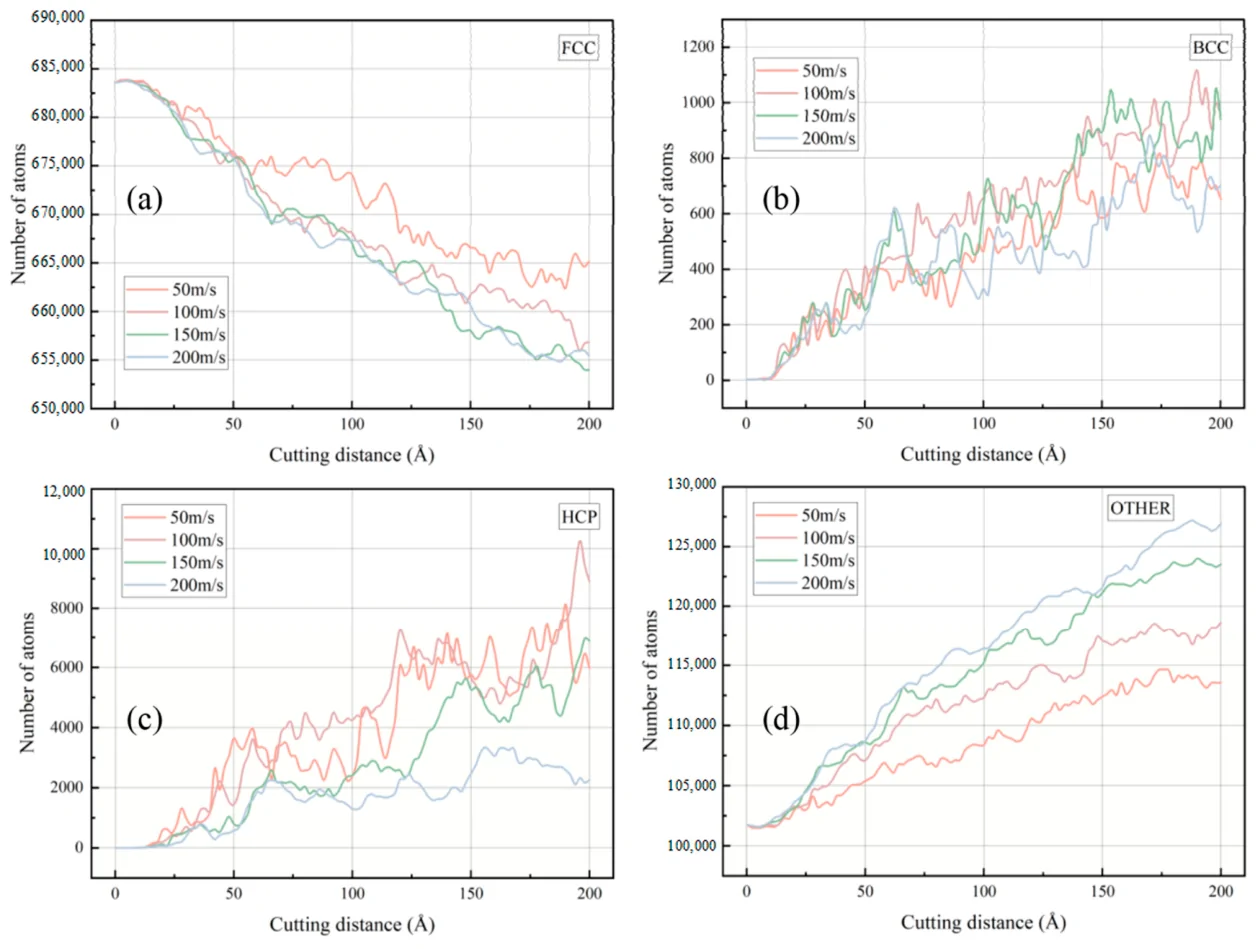
Atomic structure evolution under different cutting speeds. (**a**) FCC structures, (**b**) BCC structures, (**c**) HCP structures, and (**d**) other/disordered atomic environments.

**Figure 15:**
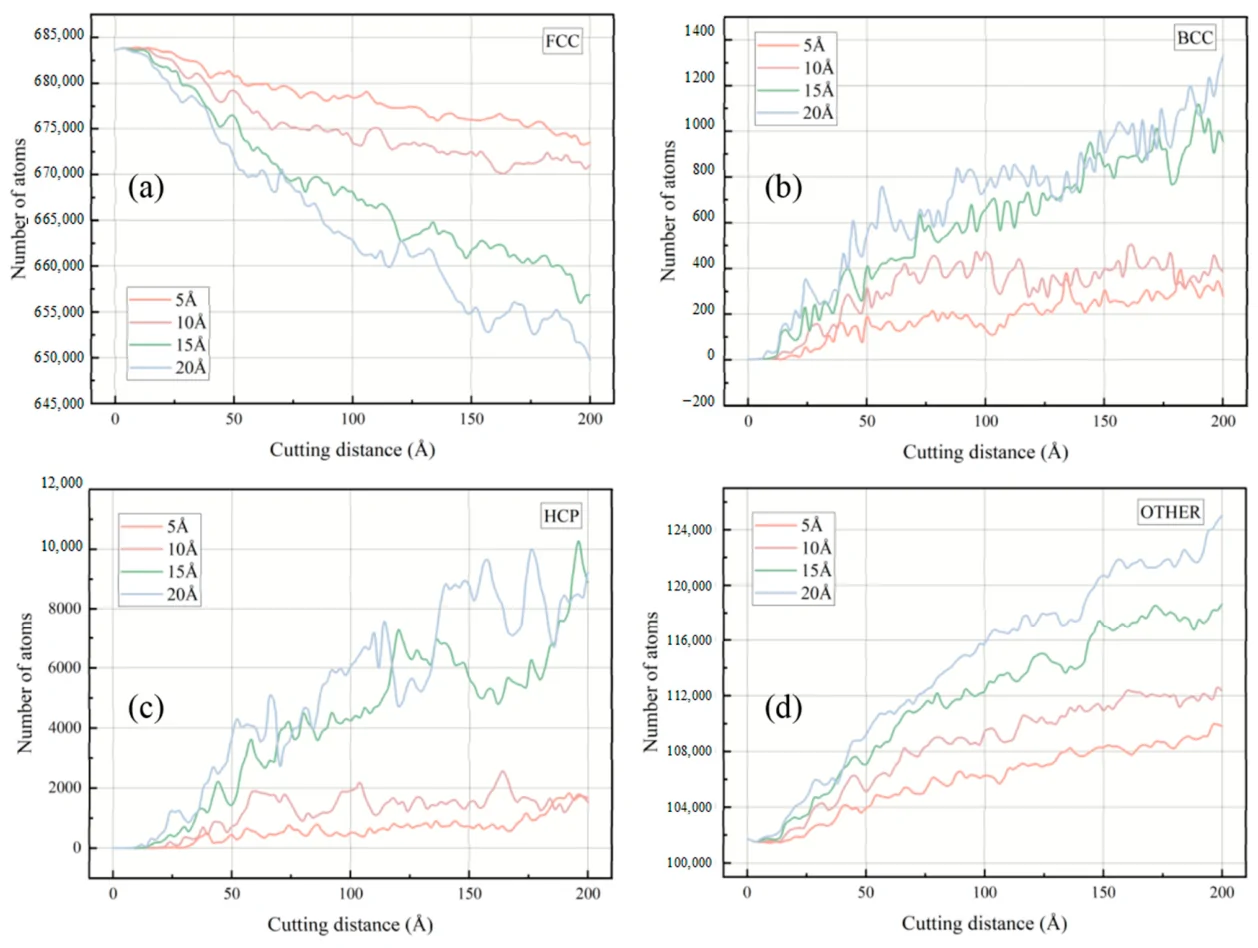
Atomic structure evolution under different cutting depths. (**a**) FCC structures, (**b**) BCC structures, (**c**) HCP structures, and (**d**) other/disordered atomic environments.

**Figure 16:**
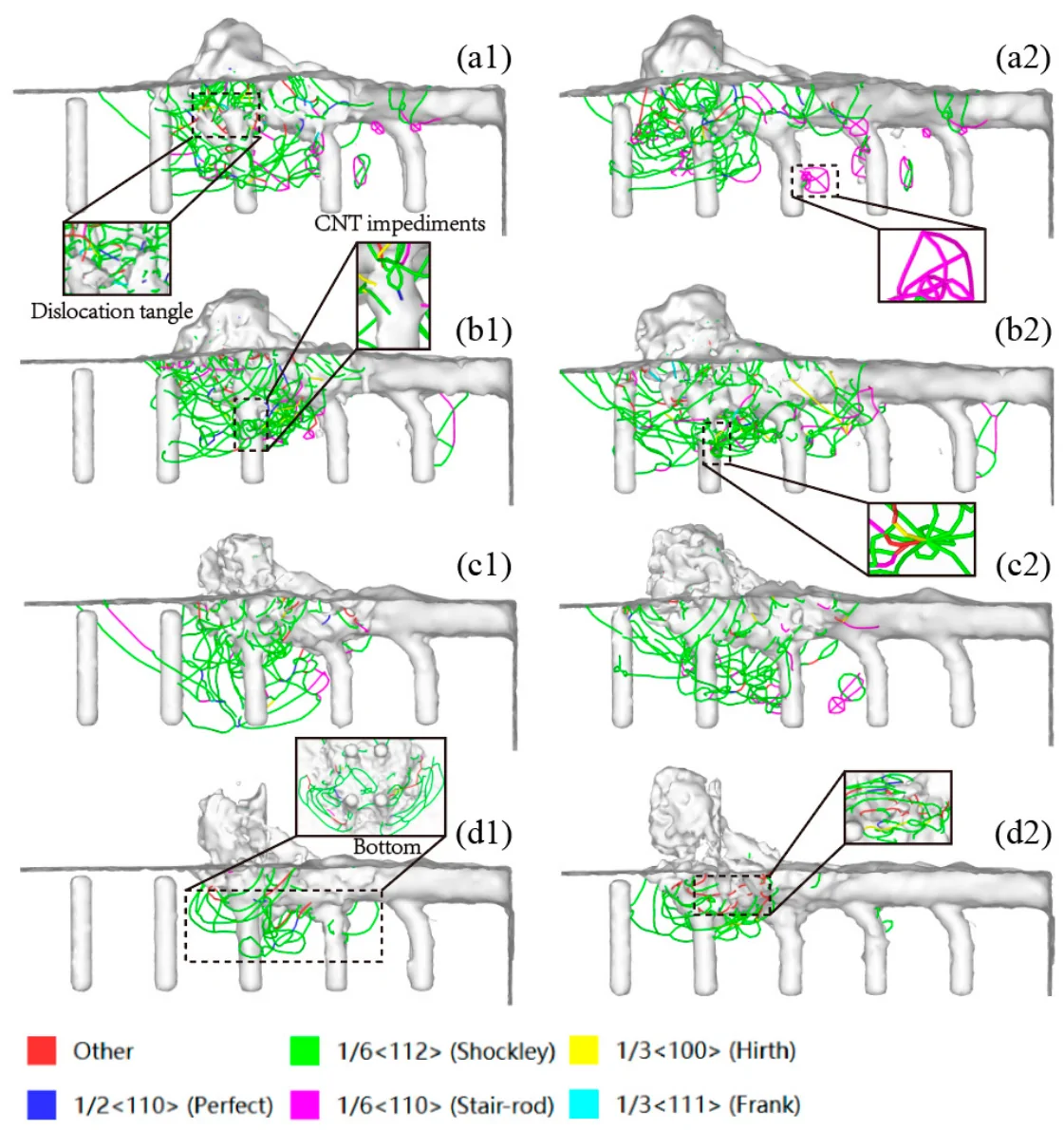
Dislocation line distribution at different cutting speeds. (**a1**) v = 50 m/s, l = 150 Å; (**a2**) v = 50 m/s, l = 200 Å; (**b1**) v = 100 m/s, l = 150 Å; (**b2**) v = 100 m/s, l = 200 Å; (**c1**) v = 150 m/s, l = 150 Å; (**c2**) v = 150 m/s, l = 200 Å; (**d1**) v = 200 m/s, l = 150 Å; (**d2**) v = 200 m/s, l = 200 Å.

**Figure 17:**
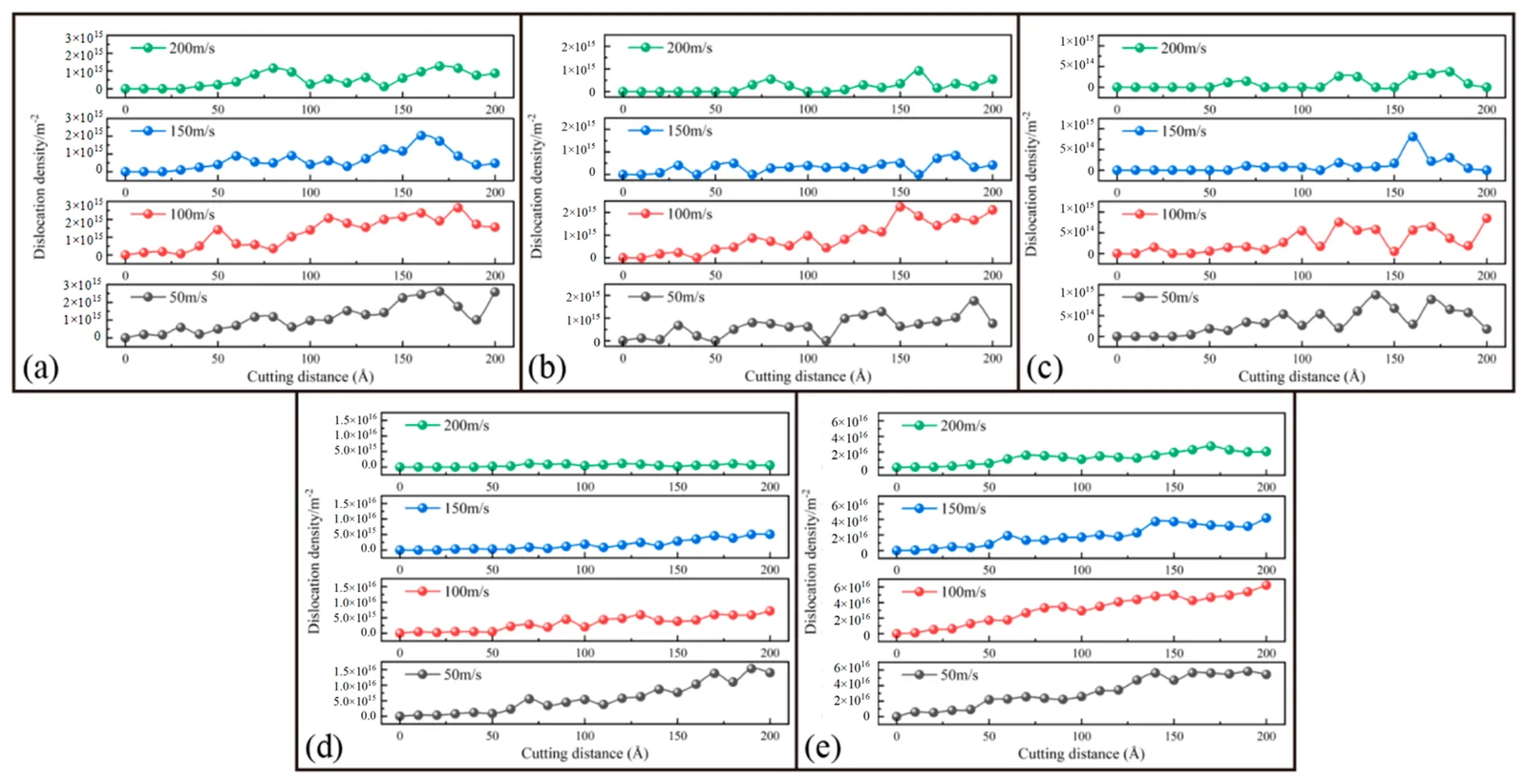
Variation in dislocation density with cutting distance at different cutting speeds. (**a**) 1/2<110> (Perfect), (**b**) 1/3<100> (Hirth), (**c**) 1/3<111> (Frank), (**d**) 1/6<110> (Stair-rod), and (**e**) 1/6<112> (Shockley).

**Figure 18:**
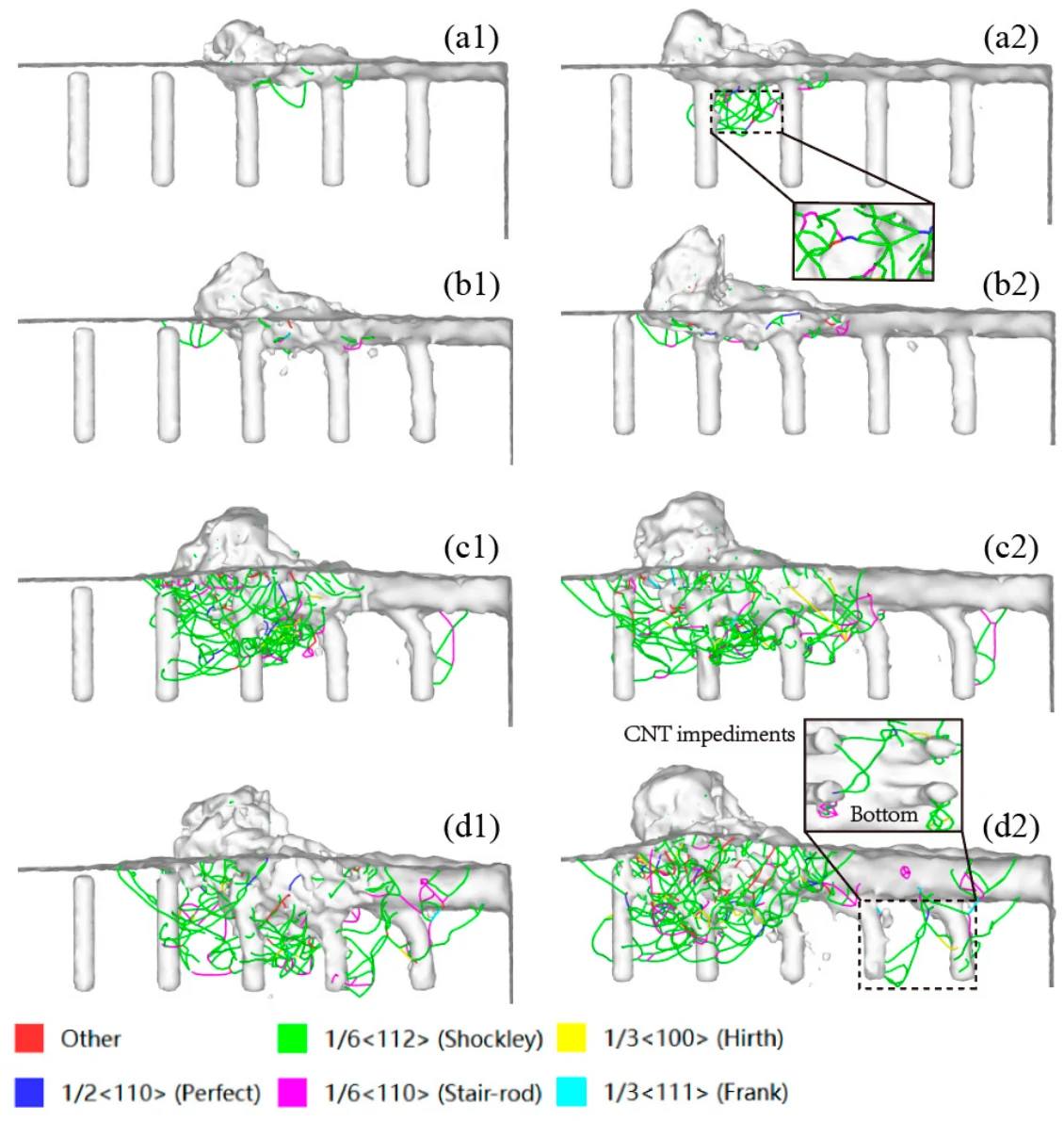
Dislocation line distribution at different cutting depths. (**a1**) ap = 5 Å, l = 150 Å; (**a2**) ap = 5 Å, l = 200 Å; (**b1**) ap = 10 Å, l = 150 Å; (**b2**) ap = 10 Å, l = 200 Å; (**c1**) ap = 15 Å, l = 150 Å; (**c2**) ap = 15 Å, l = 200 Å; (**d1**) ap = 20 Å, l = 150 Å; (**d2**) ap = 20 Å, l = 200 Å.

**Figure 19:**
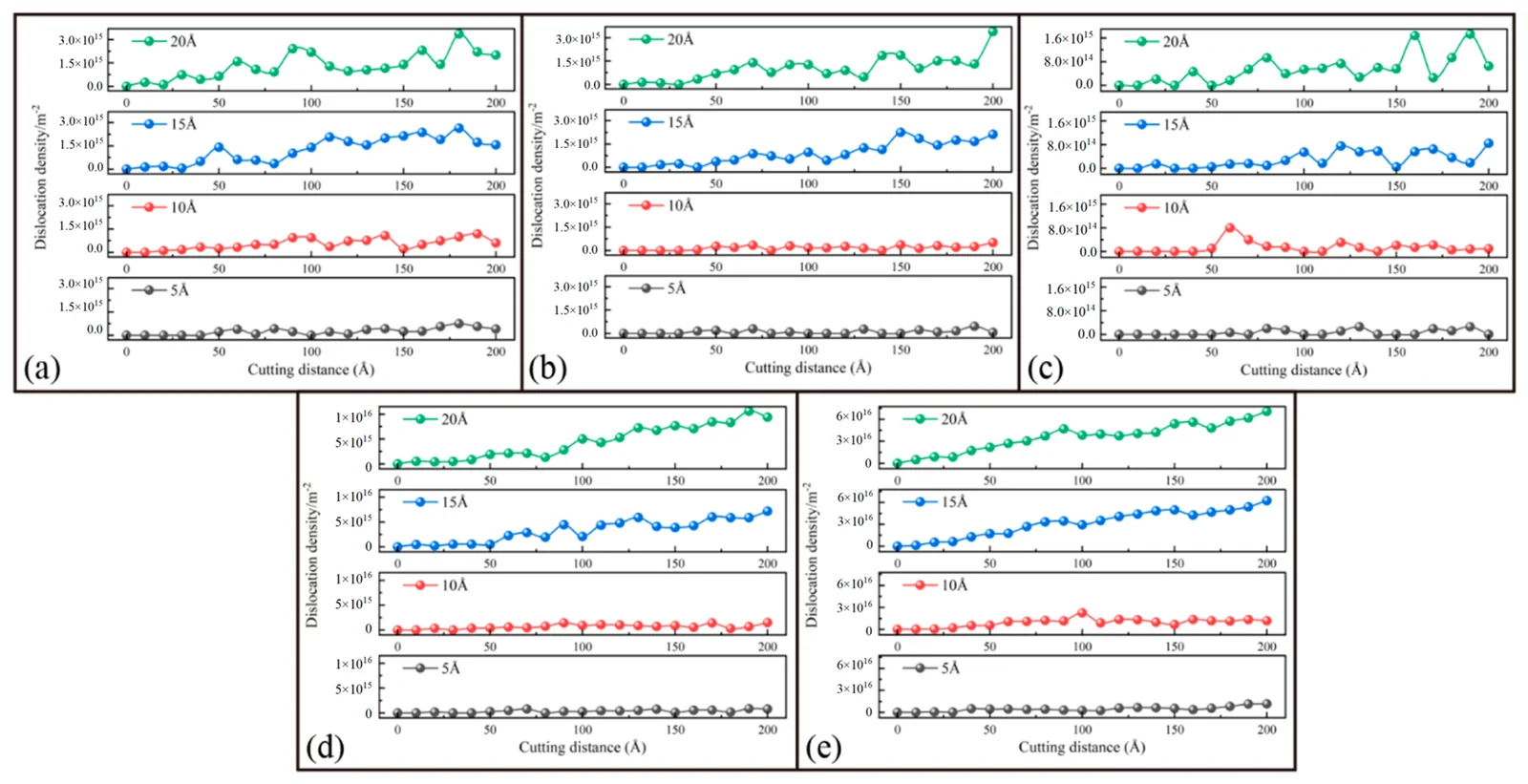
Variation in dislocation density with cutting distance at different cutting depths. (**a**) 1/2<110> (Perfect), (**b**) 1/3<100> (Hirth), (**c**) 1/3<111> (Frank), (**d**) 1/6<110> (Stair-rod), and (**e**) 1/6<112> (Shockley).

**Table 1:** Parameters in nano-cutting MD simulation.

Simulation Parameters	Value
Workpiece	CNT-Ni nanocomposites
Tool	Diamond
Model dimension	303 Å × 161 Å × 161 Å
Rake angle of the tool	0°
Clearance angle of the tool	15°
Radius of edge (r_oe_)	20 Å
Cutting speeds	50 m/s, 100 m/s, 150 m/s, 200 m/s
Cutting depth	5 Å, 10 Å, 15 Å, 20 Å
Cutting direction	(001) $\bar{1}00$
Equilibration temperature	293 K
Cutting distance	200 Å
Timestep	1 fs

**Table 2:** Potential function parameters.

Atomic Interaction	Potential	Parameters	Reference
Ni–Ni	EAM	/	[30]
Tool C–C	Tersoff	/	[31]
CNT C–C	AIREBO	/	[32]
CNT C–Ni	Lennard–Jones	*ε*_Ni-C_ = 0.02551 eV; *σ*_Ni-C_ = 2.8535 Å	[14]
Tool C–Ni	Morse	D = 1.009392 eV; r_0_ = 2.61994 Å; α = 1.9875 Å^−1^	[35]
Tool C–CNT C	Lennard–Jones	*E*_C-C_ = 2.84 × 10^−3^ eV; *σ*_C-C_ = 3.4 Å	[36]

## Data Availability

On behalf of all authors, I confirm that all data are true and reliable. Complete raw simulation data including atomic trajectory files, cutting force/temperature output records, von Mises stress and shear strain calculation results, subsurface defect statistics and dislocation density datasets are preserved in the research team’s dedicated storage equipment. All critical simulation setup information (workpiece size, cutting parameters, multi-type interatomic potential parameters for Ni, CNT, diamond tool) are comprehensively provided in Table 1 and Table 2 of this paper, supporting the repeatability of the nanocutting simulation of CNT-reinforced Ni matrix composites. The open-source software Atomsk, LAMMPS and OVITO utilized for model construction, simulation execution and data visualization are publicly available. Raw data can be obtained from the corresponding author upon reasonable request after publication.
